# CeO_2_-Supported Single-Atom Cu Catalysts Modified with Fe for RWGS Reaction: Deciphering the Role of Fe in the Reaction Mechanism by In Situ/Operando Spectroscopic Techniques

**DOI:** 10.1021/acscatal.4c01493

**Published:** 2024-07-05

**Authors:** Abdallah I. M. Rabee, Hayder Abed, Thanh Huyen Vuong, Stephan Bartling, Laura Kraußer, Hanan Atia, Nils Rockstroh, Evgenii V. Kondratenko, Angelika Brückner, Jabor Rabeah

**Affiliations:** † Leibniz-Institut für Katalyse, Albert-Einstein-Str. 29A, 18059 Rostock, Germany; ‡ Chemistry Department, Faculty of Science, Minia University, 61519 El-Minia, Egypt; § Department Life, Light and Matter, University of Rostock, Albert-Einstein-Str. 25, 18059 Rostock, Germany; ∥ State Key Laboratory of Low Carbon Catalysis and Carbon Dioxide Utilization, Lanzhou Institute of Chemical Physics (LICP), Chinese Academy of Sciences, 730000 Lanzhou, P. R. China

**Keywords:** reverse water–gas shift, CO_2_ hydrogenation, ceria, in situ/operando spectroscopy, oxygen vacancy

## Abstract

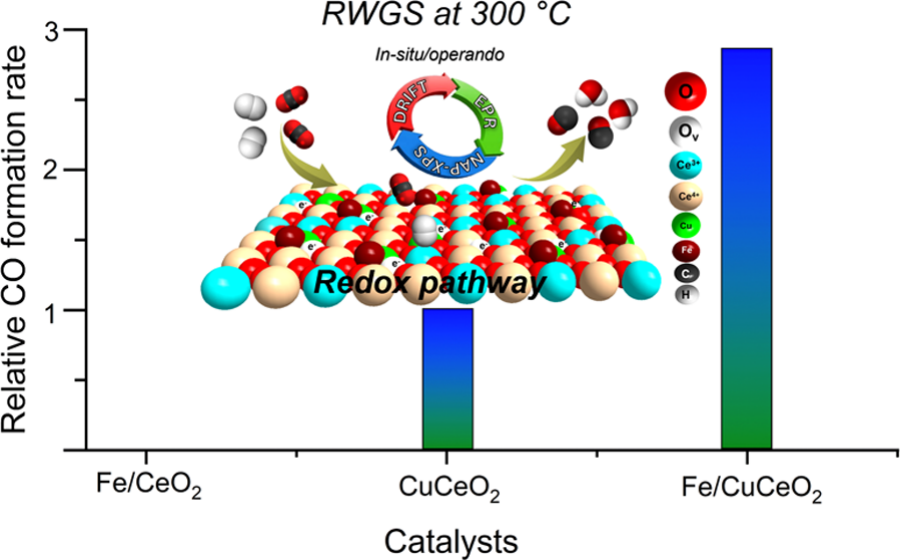

Reverse water–gas shift (RWGS) reaction has attracted much attention as a potential approach for CO_2_ valorization via the production of synthesis gas, especially over Fe-modified supported Cu catalysts on CeO_2_. However, most studies have focused solely on investigating the RWGS reaction over catalysts with high Cu and Fe loadings, thus leading to an increase in the complexity of the catalytic system and, hence, preventing the gain of any reliable information about the nature of the active sites and reaction mechanism. In this work, a CeO_2_-supported single-atom Cu catalyst modified with iron was synthesized and evaluated for the RWGS reaction. The catalytic results reveal a significant synergistic effect between CuCeO_2_ and Fe, demonstrating an activity up to three times higher than the combined catalytic activities of monometallic catalysts (Fe/CeO_2_ + CuCeO_2_) under identical conditions. Various ex situ and in situ/operando techniques are employed to unveil the concealed role of Fe in catalyst activity enhancement. The combined findings from hydrogen temperature-programmed reduction (H_2_-TPR) and operando electron paramagnetic resonance spectroscopy (EPR) reveal that the added Fe predominantly interacts with Cu-containing surface sites, resulting in the stabilization of higher proportions of Cu single sites. Near-ambient pressure X-ray photoelectron spectroscopy (NAP-XPS) and operando EPR results unveil a synergistic interplay of Fe with Cu-containing sites and CeO_*x*_ domains, efficiently enhancing both the reoxidation of Cu^+^ in Cu^+^–O_v_–Ce^3+^ moieties and the reducibility of Ce^4+^ in CeO_*x*_ domains under RWGS conditions. Detailed mechanistic studies reveal that the RWGS reaction predominantly proceeds via the redox mechanism.

## 1 Introduction

CO_2_ hydrogenation to CO via the reverse water–gas shift (RWGS) reaction is a promising process for CO_2_ utilization. This is because the resulting product, CO, can be used as a feedstock in the synthesis of valuable chemicals.^1,2^ Copper supported on CeO_2_ is one of the most promising nonprecious metal-based catalysts for this reaction due to the high activity and selectivity at atmospheric pressure.^3−9^ The addition of iron to supported Cu-based catalysts has been widely shown to improve catalytic activity and stability.^10−17^ For instance, addition of Fe on Cu/SiO_2_ enhanced the catalytic activity, by inhibiting the oxidation of Cu,^18,19^ through the rapidly spilled over oxygen released from the dissociation of CO_2_ on Cu to Fe surfaces to form iron oxides (FeO_*x*_), thus maintaining Cu in its reduced state.^18,19^ In contrast, in other studies by Reina et al.,^10,17^ the strong Cu–Fe interaction on Cu–Fe/CeO_2_–Al_2_O_3_ was found to enhance the reduction of Fe species. The enhanced activity of Fe–Cu bimetallic catalysts has been attributed not only to the synergistic effect between the two metals but also to the creation of new catalytically active sites (e.g., Cu–FeO_*x*_).^20,21^ In addition to the enhanced activity, adding Fe also inhibits the sintering of Cu particles, resulting in high stability. Moreover, it has also been noticed that the majority, if not all, of the studies have focused on investigating the high-temperature RWGS reaction (>400 °C).^11,15,17,18,22^ By comparing the activities of the Fe–Cu bimetallic catalysts from literature, it was noted that the total catalytic activity tends to approximate the sum of the activities of the respective monometallic catalysts with identical metal loading at elevated temperatures, suggesting a minor influence of synergism between the two metals in the catalytic process.^18^

Several mechanisms have been suggested for the RWGS reaction over CeO_2_-based catalysts, encompassing the direct dissociation pathway (redox mechanism) and the hydrogen-assisted pathway (associative mechanism). The latter involves intermediates like carbonate (CO_3_^2–^), formate (HCOO^–^), and carboxylate. For instance, Rodriguez et al. proposed bidentate carbonate (b-CO_3_^2–^)^3^ and bidentate formate (b-HCOO^–^) as active intermediates on Cu/CeO_2_.^3,4^ Similarly, Zhang et al.^23^ found that surface oxygen vacancies on Cu/CeO_2_ directly participate in CO_2_ adsorption and activation via forming these bidentate species as key intermediates.^23^ Hess et al. reported that the reaction over the Cu/CeO_2_ catalysts with low Cu loading involves both redox and associative mechanisms, with the former identified as the primary pathway.^24^ In the associative mechanism, formate and carbonate species serve as intermediates.^24^ The same carbonate species are also suggested by Goguet et al.^25^ as intermediates over a different CeO_2_-based catalytic system, such as Pt/CeO_2_, using a combination of steady-state isotopic transient kinetic analysis (SSITKA) and diffuse reflectance infrared Fourier transform spectroscopy (DRIFTS) analysis. This may suggest that the ceria support plays the main role in tailoring adsorbed reactive species, regardless of the nature of the metal, while the role of the metal is to activate H_2_ and generate oxygen vacancies.^25^ Goguet et al.^25^ draw their conclusions based on the fast ^13^C exchange of carbonates compared with formate when the reaction mixture was switched from ^12^CO_2_ + H_2_ to ^13^CO_2_ + H_2_. However, Jacobs et al.^26^ showed that upon addition of water to the reaction mixture, the exchange rate of formates is the same as the carbonate exchange rate over the Pt/CeO_2_ catalyst. They also found that carbonates exchange rapidly even in the absence of H_2_. They reported that the rapid exchange of surface carbonates is not solely attributed to the reaction process but rather to other factors, such as thermal desorption.^26^ Consequently, these studies suggest that identifying the reaction mechanisms of the RWGS reaction over CeO_2_-based catalysts remains a subject of intense debate. The focus on catalysts with high metal loadings, containing various coexisting metal species such as single sites, clusters, and nanoparticles in most of the preceding studies,^11,15,17,18,22^ adds complexity to the catalytic system, making it challenging to gain reliable insights into the underlying reaction mechanisms.

Therefore, this study is specifically designed to investigate the highly debated reaction mechanism using a copper single-atom catalyst, with a particular emphasis on unraveling the fundamental aspects of the synergistic interaction between Cu and Fe, as well as the role of Fe in the reaction mechanism. To this end, unmodified and iron-modified CeO_2_-supported single-atom Cu catalysts have been prepared and subjected to rigorous examination using cutting-edge techniques, such as near-ambient pressure X-ray photoelectron spectroscopy (NAP-XPS), in situ DRIFTS, and in situ electron paramagnetic resonance spectroscopy (EPR).

## 2 Materials and Methods

### 2.1 Catalyst Preparation

Cu-doped CeO_2_ (5 g), denoted as CuCeO_2_ hereafter, was synthesized using a coprecipitation method. 12.55 g of Ce(NO_3_)_3_·6H_2_O (Sigma-Aldrich Chemicals, 99.0%) and 0.095 g of Cu(NO_3_)_2_·6H_2_O (Sigma-Aldrich Chemicals, >99.0%) for 0.5 wt % Cu were dissolved in 500 mL of deionized water, followed by stirring for 15 min and subsequent addition of 1 M NaOH (Fisher Chemical, > 99.1%) until reaching pH 8.5. This solution was stirred for 3 h, and the resulting slurry was filtered, washed with deionized water, and dried at 100 °C for 24 h. The obtained solid was ground to a fine powder and calcined at 600 °C (heating rate of 2 °C min^–1^) for 3 h in static air. The bare CeO_2_ support was also synthesized using the same protocol, excluding the addition of the Cu precursor.

The iron-modified CuCeO_2_ catalyst was synthesized using the conventional wet impregnation method with a nominal iron loading of 0.3 wt %. To achieve this, 0.065 g of Fe(NO_3_)_3_.9H_2_O (Sigma-Aldrich Chemicals, ≥98.0%) was dissolved in 40 mL of deionized water. Subsequently, 3 g of CuCeO_2_ powder was added at room temperature and left under constant stirring for 3 h. Following this, the slurry was dried at 100 °C for 24 h to remove all water. The dried sample was then ground to a fine powder using a mortar and pestle and calcined at 500 °C (heating rate of 2 °C min^–1^) for 3 h in static air. A control sample without Cu was prepared using a similar approach by impregnating the CeO_2_ support with a Fe(NO_3_)_3_·9H_2_O solution in deionized water to achieve a 0.3 wt % Fe loading. The CuCeO_2_ and CeO_2_ samples impregnated with Fe are denoted hereafter as Fe/CuCeO_2_ and Fe/CeO_2_, respectively.

### 2.2 Catalyst Characterization

Prior to characterization, the catalysts were reduced at 400 °C in a 50 vol % H_2_/N_2_ atmosphere for 2 h, with a total flow rate of 13 mL min^–1^. The inductively coupled plasma optical emission spectroscopy (ICP-OES) method was employed to determine the bulk elemental composition of Cu and Fe. The measurements were conducted using a 715-ES ICP emission spectrometer (Varian, Palo Alto, CA). Before analysis, the samples were dissolved in a mixture of HF and aqua regia at 200 °C and 60 bar through microwave-assisted digestion. Brunauer–Emmett–Teller (BET) surface area, pore volume, and average pore diameter measurements were carried out by N_2_ adsorption at 77 K using a Micromeritics ASAP 2010 instrument. Typically, 100 mg of sample was placed in the analysis tube and degassed at 200 °C for 4 h prior to exposure to N_2_ gas. The powder X-ray diffraction (XRD) characterizations of all samples were carried out on a Panalytical X’Pert PRO diffractometer equipped with an X’Celerator RTMS detector using Ni-filtered Cu–Kα radiation (λ = 0.154 nm) operating at 40 kV and 40 mA. Data were collected stepwise (0.021° s^–1^) in the range of 10–80° (2θ) with a divergence slit of 2°. Peak positions and profiles were fitted with Pseudo-Voigt functions using the HighScore Plus software package (Panalytical). Phase identification was done using the PDF-2 database of the International Center of Diffraction Data (ICDD). Hydrogen temperature-programmed reduction (H_2_-TPR) measurements were conducted using a Micromeritics Autochem II 2920 instrument. In a typical experiment, the catalyst (100 mg) was pretreated in a flow of synthetic air (50 mL·min^–1^) at 400 °C for 30 min. The sample was then flushed under Ar for 30 min and cooled to room temperature (RT). After reaching RT, the catalyst was exposed to a 10% H_2_/Ar flow (50 mL·min^–1^) while the temperature was ramped up to 900 °C at a rate of 10 °C min^–1^. H_2_ consumption was monitored using a thermal conductivity detector (TCD). Calibration of the TCD through TPR of CuO facilitated the quantitative evaluation of H_2_ consumption. Scanning transmission electron microscopy (STEM) measurements were carried out utilizing a probe aberration-corrected JEM-ARM200F (JEOL, Corrector: CEOS) operating at 200 kV. This microscope is equipped with a JED-2300 (JEOL) energy-dispersive X-ray spectrometer (EDX) having a silicon drift detector (dry SD60GV) and an Enfinium ER (Gatan) electron energy-loss spectrometer (EELS). For STEM imaging, both high-angle annular dark field (HAADF) and annular bright field (ABF) detectors were employed, while an annular dark field (ADF) detector was used for position control for EELS acquisition. Dual EELS was performed at a camera length of 4 cm, an illumination semi-angle of 27.8 mrad, and a filter entrance aperture semi-angle of 41.3 mrad using the low loss region for compensation of energy shifts during acquisition. The solid samples were deposited onto a holey carbon-supported Ni grid (mesh 300) without any pretreatment and subsequently transferred to the microscope for analysis.

Electron paramagnetic resonance (EPR) measurements were performed using an X-band cw-spectrometer ELEXSYS 500-10/12 (Bruker) with a microwave power of 6.3 mW, a modulation frequency of 100 kHz, and a modulation amplitude up to 5 G. Operando EPR experiments were carried out in a homemade quartz plug-flow reactor (3.0 mm inner diameter and 0.5 mm wall thickness). The reactor was connected to a gas-dosing device with mass-flow controllers (Bronkhorst) at the inlet and a quadrupole mass spectrometer (Omnistar, Pfeiffer Vacuum GmbH) at the outlet for online product analysis. For each operando EPR experiment, 50 mg of the catalyst mixed with 50 mg of quartz and a total feed gas flow of 25 mL·min^–1^ were used. Initially, the catalyst was reduced in flowing 50% H_2_/Ar at 300 °C for 45 min, followed by flushing with Ar at 25 °C, and spectra were recorded at both 25 and −173 °C. Then, the pre-reduced catalysts were exposed to the following sequence: (1) heating at 300 °C in pure CO_2_ for 21 min, cooling to 25 °C under continuous flow of CO_2_, flushing at 25 °C with 25 mL·min^–1^ of Ar for 15 min, and recording spectra at 25 and −173 °C; (2) re-reduction at 300 °C in pure H_2_ for 15 min, exposure to a flow of CO_2_ + H_2_ in Ar (CO_2_/H_2_ ratio = 1/3) at 300 °C for 1 h, cooling to 25 °C under a continuous flow of CO_2_ + H_2_, then flushing with 25 mL·min^–1^ of Ar for 15 min at 25 °C, and recording spectra at 25 and −173 °C. During these experiments, different gas concentrations were measured by online mass spectrometry. The EPR spectra of Cu-containing catalysts were simulated using the software package Easyspin implemented in MATLAB program.^27^

To detect O_2_^•–^ and CO_2_^•–^ radicals, additional EPR experiments were carried out using 5,5-dimethyl-1-pyrroline-*N*-oxide (DMPO) as a spin trap. In each experiment, a mixture of 1 mL anhydrous cyclohexane and 20 μL DMPO was prepared under anaerobic conditions. For the detection of O_2_^•–^ radicals, about 10 mg of the fresh catalysts CuCeO_2_ and Fe/CuCeO_2_ were used. For each sample, 200 μL of DMPO in cyclohexane was injected into an EPR tube containing 10 mg of catalyst for recording EPR spectra at room temperature. For the detection of CO_2_^•–^ radicals, 25 mg of Fe/CuCeO_2_ was placed in an evacuatable EPR tube. The catalyst was heated up to 300 °C under evacuation, then exposed to a flow of pure H_2_ at 300 °C for 5 min, and evacuated to remove the produced H_2_O inside the tube. This process was repeated 3 times followed by exposure to a flow of pure CO_2_ 3 times. After cooling down to room temperature, 200 μL of DMPO in cyclohexane was loaded into the evacuated EPR tube containing the catalyst under a CO_2_ atmosphere.

In situ diffuse reflectance infrared Fourier transform spectra (DRIFTS) were collected on a Nicolet 6700 FTIR spectrometer using a high-temperature Praying Mantis reaction cell (Harrick) with CaF_2_ windows, equipped with a temperature control unit (Eurotherm). Each spectrum was obtained by averaging 64 scans recorded with a resolution of 4 cm^–1^. Typically, ca. 50 mg of the as-synthesized catalyst powder was deposited on a layer of 70 mg quartz. First, the cell was flushed with He (13 mL min^–1^) while the temperature was increased to 400 °C at a rate of 10 °C min^–1^. Subsequently, the catalyst surface was reduced for 120 min under a flow of 50% mixture of H_2_ in He (13 mL·min^–1^) at 400 °C. Thereafter, the cell was flushed with He (13 mL·min^–1^) at 400 °C, and background spectra were recorded. The reaction was performed in a temperature range between 350 and 400 °C with a mixture of H_2_/CO_2_/He = 3:1:2 at a total flow rate of 18 mL·min^–1^. An additional RWGS experiment at 400 °C was also conducted over a selected catalyst under identical conditions. However, in this experiment, the reduced catalyst was first exposed to a flow of H_2_/CO_2_/He = 3:1:2 (18 mL min^–1^) for 15 min and then switched to D_2_/CO_2_/He = 3:1:2 (18 mL min^–1^) for another 15 min. The intensity of the signals is given in a log(1/*R*) scale, where *R* = *I*_r_/*I*_b_ represents the ratio between the single beam spectra of the sample at reaction conditions and the single beam spectra of the sample before reaction at a specific temperature.

A series of in situ DRIFTS experiments involving the switching between a CO_2_/He mixture (1:5) and a H_2_/He mixture (3:3) were conducted at 400 °C. Before spectral acquisition, the catalyst was reduced in 50% H_2_ in He (13 mL·min^–1^) for 2 h at 400 °C. Subsequently, it was thoroughly purged with He for 6 h at the same temperature and left under a He flow for 10 h at room temperature to minimize residual adsorbed H_2_. Following this, the temperature was increased to 400 °C under a He flow. Once the desired temperature was reached, the catalysts were exposed to a flow of 16.6 vol % CO_2_ in He (18 mL·min^–1^) for 10 min before switching to 50% H_2_ in He (18 mL min^–1^).

For CO adsorption experiments, the catalyst sample was purged with He while heating to 400 °C, after which the gas mixture was switched to 50% H_2_ in He at a flow rate of 13 mL min^–1^ for 2 h. After reduction, the sample was purged with He gas at 400 °C for 30 min and subsequently cooled to room temperature under He with a flow rate of 13 mL min^–1^. A background spectrum was then recorded. Subsequently, a 2% CO in He gas mixture was introduced into the cell for 30 min at a flow rate of 13 mL min^–1^, during which spectra were collected every 5 min. While continuing to collect spectra, the sample cell was then purged with He at a flow rate of 13 mL min^–1^ for 10 min. Similar CO adsorption experiments were performed in which CO_2_ flowed after the reduction step at 400 °C for 15 min, followed by the identical previous procedure. Each CO-DRIFTS spectrum was recorded at 25 °C.

Operando near-ambient pressure X-ray photoelectron spectroscopy (NAP-XPS) studies were conducted using a laboratory NAP-XPS system (SPECS Surface Nano Analysis GmbH, Germany). The setup was equipped with a differentially pumped Phoibos 150 electron energy analyzer with a nozzle diameter of 500 μm, a monochromated Al Kα radiation source (*E* = 1486.6 eV), and a laser system for sample heating. Gases were introduced into the analysis chamber via mass-flow controllers (Brooks, GF40) at a total pressure of 2 mbar. Reactants and products were monitored using a quadrupole mass spectrometer (QMS, MKS e-vision 2) attached to the lens system of the spectrometer. Powder samples were compressed into 5 mm diameter tablets on a stainless-steel sample plate using a laboratory press and a load of approximately 1 t. Temperature was monitored by a thermocouple attached to a sample plate pressed onto the sample surface. The electron binding energies were referenced to the C 1s core level of native carbon species (C–C and C–H bonds) at an energy of 284.8 eV. To analyze the recorded XPS spectra, peak deconvolution was performed using Gaussian–Lorentzian curves with the Unifit 2023 software.

### 2.3 Catalytic Tests

The performance of the catalysts was assessed in a quartz fixed-bed reactor at atmospheric pressure over a temperature range of 300–400 °C. Typically, 50 mg of the sample with a particle size of ca. 250–315 μm diluted with 450 mg of quartz sand was placed inside the reactor. Prior to exposure to the reactant gas mixture (39% H_2_, 13% CO_2_, and 45% N_2_), the catalyst was reduced for 120 min under a flow of 50% H_2_ in He (13 mL·min^–1^) at 400 °C. The catalytic tests were conducted with a total flow of 30 mL·min^–1^ and a gas hourly space velocity (GHSV) of 36,000 mL·g_cat_^–1^·h^–1^. The flow rate was controlled using mass-flow controllers (Bronkhorst). Each gas had a purity of more than 99.99%. The effluent gases were analyzed using an online gas chromatograph (Shimadzu 2014) equipped with thermal conductivity and FID detectors. Two columns were used for gas separation and analysis: one for the separation of CO, O_2_, and H_2_ (Molsieve 5 Å, Agilent) and the other for the separation of CO and CO_2_ (PoraPLOT Q, Agilent). The conversion of CO_2_ was calculated from the inlet and outlet molar flow rates, as shown in eq 1. The selectivity to CO was calculated using eq 2. The rates of CO formation (*r*_CO_) normalized by the total catalyst mass were calculated using eq 3.
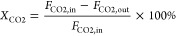
1
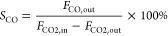
2
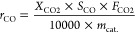
3where *X*_CO2_ is the CO_2_ conversion (%), *S*_CO_ is the selectivity to CO (%), *F*_CO_ and *F*_CO2_ are the molar flow rates of CO and CO_2_, respectively, and *m*_cat._ is the catalyst mass.

## 3 Results and Discussion

### 3.1 Catalyst Characterization

Table S1 presents the chemical composition and textural properties of the catalysts. The Cu and Fe contents closely align with the nominal values. The XRD patterns of all samples are virtually identical, showing exclusively the presence of the cubic CeO_2_ phase (JCPDS 01-079-6884, Figure S1). Moreover, all catalysts are mesoporous as evidenced by similar type-IV adsorption–desorption isotherms (IUPAC) and mean pore diameters (Figure S2), and their BET surface areas do not differ much as well.

**Figure 1:**
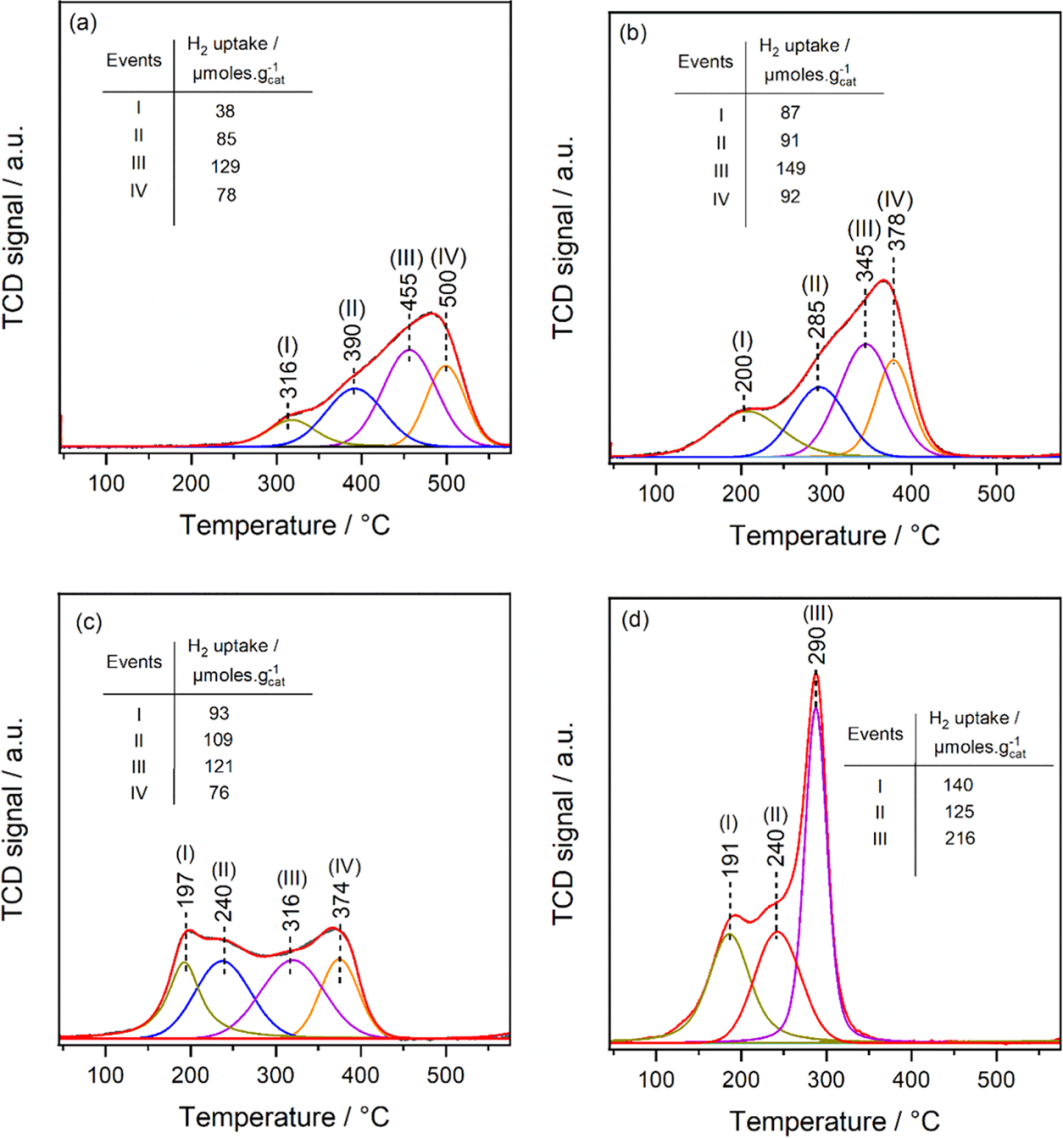
Deconvoluted H_2_-TPR profiles recorded as a function of temperature for the low-temperature reduction zone of (a) CeO_2_ (b) Fe/CeO_2_ (c) CuCeO_2_, and (d) Fe/CuCeO_2_.

The catalysts’ reducibility was investigated using H_2_-TPR, revealing distinct low-temperature (150–570 °C) and high-temperature (>570 °C) regions associated with surface Ce^4+^ and bulk Ce^4+^ reduction, respectively (Figures S3, S4, and Table S2).^28^ Deconvoluted TPR profiles for the low-temperature region, along with corresponding H_2_ consumption (in μmoles·g_cat_^–1^), are depicted in Figure 1. The TPR profile of bare CeO_2_ (Figure 1a) reflects surface heterogeneity, with a total H_2_ consumption of 330 μmoles·g_cat_^–1^. Addition of Fe on CeO_2_ (Figure 1b) significantly lowers the reduction temperature of surface Ce^4+^ ions (Figure S4). The facile reduction of Fe/CeO_2_ is attributed to the formation of easily reducible Fe^3+^–O^2–^–Ce^4+^ species,^29^ with a hydrogen consumption of 419 μmoles·g_cat_^–1^ in the low-temperature region (Table S1). Assuming all Fe is present as Fe_2_O_3_, this aligns closely with the required hydrogen for surface Ce^4+^ reduction (330 μmoles·g_cat_^–1^) and Fe_2_O_3_ reduction (Fe^3+^ → Fe^0^, 80.6 μmoles·g_cat_^–1^). Importantly, after Fe addition, H_2_ consumption during all reduction events of Fe/CeO_2_ increased (Figure 1b) compared to CeO_2_ alone, strongly suggesting its involvement in all reduction events. In the presence of 0.46 wt % Cu (CuCeO_2_), a comparable behavior is observed with four reduction events at temperatures 192, 237, 316, and 374 °C. These events are considered analogous to the four reduction events observed in the TPR profile of CeO_2_ alone at 316, 390, 455, and 500 °C. The increased H_2_ consumption during events I and II in CuCeO_2_, compared to bare CeO_2_, suggests the predominant reduction of most Cu^2+^ in the first two events, indicating the presence of two different Cu^2+^ species. Notably, the H_2_ consumption during events I and II in the TPR profile of CuCeO_2_ exceeds the amount required to completely reduce all Cu^2+^ to Cu^+^/Cu^0^, suggesting the involvement of Ce^4+^ strongly interacting with highly dispersed Cu^2+^ sites (Cu^2+^–O^2–^–Ce^4+^).^30,31^ Meanwhile, events at ≥316 °C are attributed to the reduction of surface CeO_*x*_ species (–Ce^4+^–O^2–^–Ce^4+^).^30^ The enhanced reduction of surface CeO_*x*_ domains in CuCeO_2_ at 316 and 374 °C (Figure 1c), compared to bare CeO_2_ at 455 and 500 °C (Figure 1a), can be attributed to the increased oxygen mobility following the addition of Cu.^31,32^ Alternatively, this enhancement could be due to the CeO_*x*_ domains being located in close proximity to Cu^2+^–O^2–^–Ce^4+^ ensembles, which results in a weaker interaction with Cu-containing sites.

The addition of Fe to CuCeO_2_ resulted in a significant increase in the H_2_ consumption during the first two reduction events (Figure 1d), indicating that a substantial amount of the added Fe underwent reduction during these events. This indicates that Fe preferably interacts with Cu^2+^–O^2–^–Ce^4+^ sites. The simultaneous reduction of Fe alongside Cu^2+^–O^2–^–Ce^4+^ moieties at the same temperature in events I and II (Figure 1d) suggests that they share the same (O^2–^) oxide in Cu^2+^–O^2–^–Ce^4+^ moieties. However, it is important to note that the addition of Fe has negligible effect on the reduction temperature of Cu^2+^–O^2–^–Ce^4+^ moieties (first two events) but significantly enhanced the reduction of surface CeO_*x*_ species, as evident from the merging of reduction events at 316 and 374 °C of CuCeO_2_ into a single peak at 290 °C in the TPR profile of Fe/CuCeO_2_. The associative increase in H_2_ consumption of the reduction event at 290 °C indicates that a portion of Fe also interacts with CeO_*x*_, leading to the formation of Fe^3+^–O^2–^–Ce^4+^ sites. These sites are highly reducible compared to the same site on Fe/CeO_2_, owing to the enhanced oxygen mobility in the presence of both Cu and Fe ions.^31−33^

**Figure 2:**
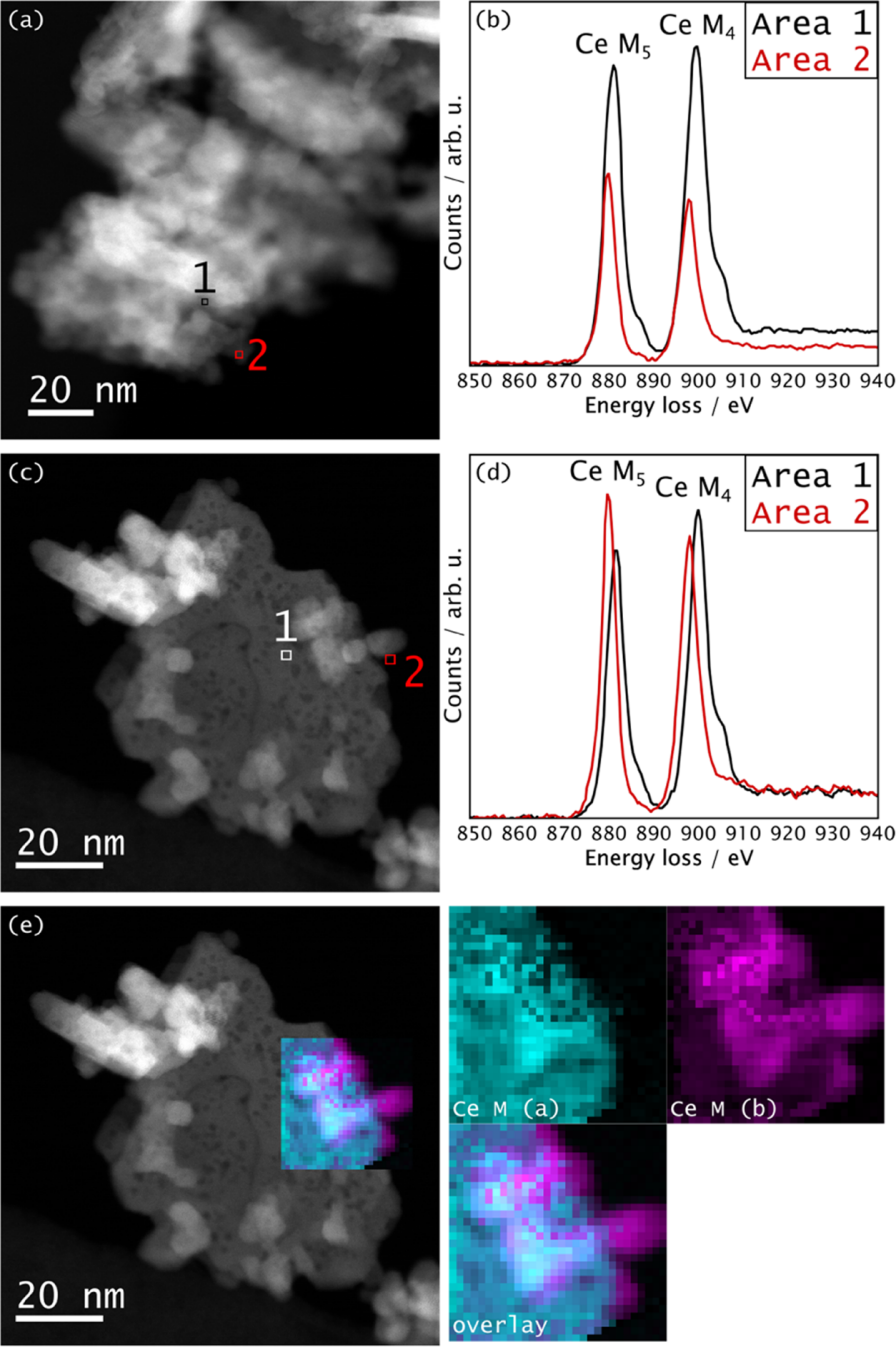
STEM-ADF images and EELS spectra of (a, b) CuCeO_2_ and (c, d) Fe/CuCeO_2_ after ex situ reduction at 400 °C for 2 h under a flow (13 mL·min^–1^) of 50% H_2_ in Ar. In (e), the STEM-ADF image of Fe/CuCeO_2_ is exhibited with an overlaid false-color elemental map. This map, as well as the single-color elemental maps on the right, illustrates the distribution of Ce^4+^ [denoted as Ce M (a) and Ce^3+^ (denoted as Ce M (b)].

The catalysts have been further characterized by STEM, EDX, and EELS. As depicted in Figure S5, HAADF images generally reveal the presence of aggregates consisting of multiple CeO_2_ crystallites. In Figure S5, the existence of pores is indicated by darker regions suggesting areas of decreased thickness. Cu and Fe species cannot be identified from HAADF images because of the brighter appearance of Ce due to its higher Z contrast. In Figure 2, EELS spectra of CuCeO_2_ and Fe/CuCeO_2_ are exhibited at different positions in the material, visualized in the corresponding HAADF images. All of the examined spectra showed the characteristic M_4_,_5_ edge of cerium, but no evidence of copper and iron could be resolved in their respective regions. The absence of copper and iron detection using EDX (Figure S6) or EELS analytical techniques across multiple images of the samples implies that these elements might either aggregate within distinct crystallites that evade observation in the STEM or exist in a highly dispersed state. The absence of diffraction patterns associated with CuO or Fe_2_O_3_ in XRD reinforces the idea that these species are more likely to be highly dispersed rather than present in separate crystalline entities. Analysis of the positions of the M_4,5_ edges in EELS spectra provides information about the valence state of Ce ions.^34,35^ The peak positions of the M_5_ and M_4_ edges of Ce^4+^ appear at higher energy loss compared to those of Ce^3+^. The shape of the spectra also offers similar insights, where the intensity ratio between M_5_ and M_4_ edges increases with the ratio of Ce^3+^/(Ce^4+^ + Ce^3+^).^36^ Furthermore, the presence of small satellite peaks in the EELS spectra of the M_5_ and M_4_ edges is a characteristic feature of Ce^4+^.^34^ In light of this, the EELS results presented in Figure 2b,d suggest that both catalysts exhibit the coexistence of Ce^3+^ and Ce^4+^ cations within the bulk of the particles, with Ce^3+^ prevailing at the edges/surface, as also visualized in Figure 2e, consistent with the findings of other research groups.^35^

### 3.2 Catalytic Performances and Kinetics

**Figure 3:**
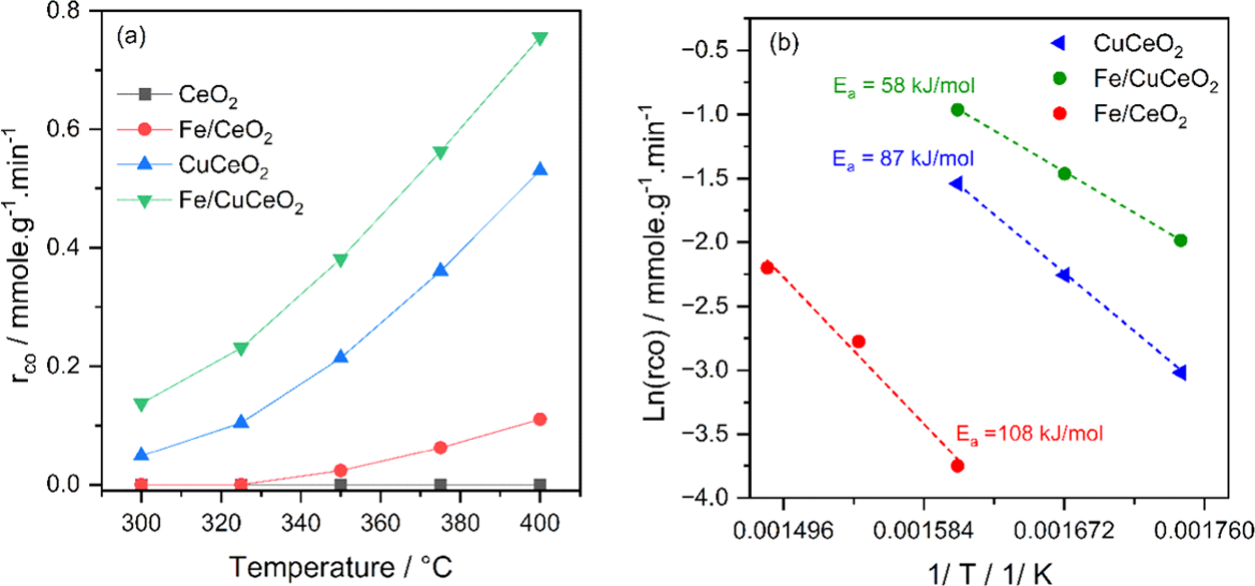
(a) CO formation rate over CeO_2_, Fe/CeO_2_, CuCeO_2_, and Fe/CuCeO_2_ catalysts; and (b) the Arrhenius plots of CO formation rates. Reaction conditions: *P* = 1 atm, CO_2_:H_2_ = 1:3, GHSV = 36,000 mL·g^–1^·h^–1^.

The catalytic activity and selectivity of bare CeO_2_ support, Fe/CeO_2_, CuCeO_2_, and FeCuCeO_2_ catalysts for the RWGS reaction were assessed over a temperature range of 300 to 400 °C under a space velocity of 36,000 mL·g_cat_^–1^·h^–1^. As shown in Figures 3a and S7, the bare support was inactive within the studied reaction temperature range. The addition of 0.3 wt % Fe on the CeO_2_ support resulted in a slight improvement in catalytic activity, with the onset temperature at around 350 °C, where the catalyst is nearly inactive at lower temperatures. In contrast, incorporating 0.4 wt % Cu in/on CeO_2_ significantly increased the catalytic activity, showing a rise in CO_2_ conversion, and thus the CO formation rate with increasing reaction temperature. Although Fe/CeO_2_ with 0.3 wt %Fe exhibits almost no activity at temperatures ranging from 300 to 350 °C, the addition of a similar amount of iron to CuCeO_2_ results in a CO_2_ conversion reaching up to three times higher than that of CuCeO_2_ within the same temperature range. This observation suggests a synergistic effect between iron and CuCeO_2_. The superior catalytic activity of the Fe/CuCeO_2_ catalyst emphasizes the crucial role of iron, particularly, at lower temperatures (≤350 °C). However, this synergistic effect becomes less pronounced at higher temperatures of 375 and 400 °C, where the overall CO_2_ conversion over Fe/CeO_2_ and CuCeO_2_ approaches that of Fe/CuCeO_2_. This is because Fe/CeO_2_ becomes an active catalyst at those temperatures. Almost all of the studies in the literature focus on high-temperature RWGS reaction (≥400 °C), where Fe has little influence on activity enhancement. This study, however, emphasizes the significant synergy between Fe and CuCeO_2_ at low temperatures. Furthermore, Fe/CeO_2_, CuCeO_2_, and Fe/CuCeO_2_ catalysts consistently demonstrated 100% CO selectivity throughout the studied range of reaction temperatures.

The Arrhenius plots for CO formation are presented in Figure 3b. For both CuCeO_2_ and Fe/CuCeO_2_ catalysts, the activation energy (*E*_a_) values were calculated within the temperature range of 300–350 °C, where the CO_2_ conversions were <10%, thus well below the equilibrium conversion as shown in Figure S7. Whereas, for the less-active Fe/CeO_2_ catalyst, the activation energy was calculated within the temperature range of 350–400 °C. For the Fe/CeO_2_ catalyst, the *E*_a_ value for CO formation was 108 kJ mol^–1^, while it was 87 kJ mol^–1^ for CuCeO_2_. The *E*_a_ for CO_2_ conversion on Fe/CuCeO_2_ (58 kJ mol^–1^) is significantly lower than that on either Fe/CeO_2_ or CuCeO_2_. Therefore, the Fe and Cu species function synergistically to reduce the activation energy for CO_2_ conversion.

### 3.3 In Situ and Operando Characterization

#### 3.3.1 In Situ DRIFTS Studies

**Figure 4:**
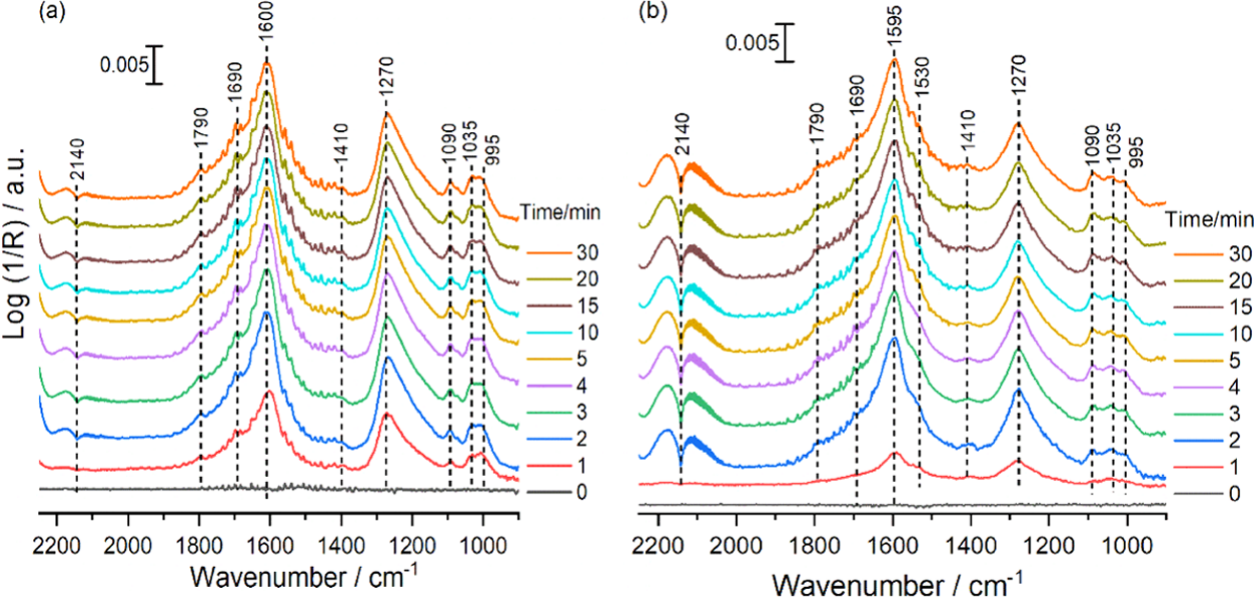
In situ time-resolved DRIFTS spectra of the RWGS reaction over (a) bare CeO_2_ and (b) Fe/CuCeO_2_. Reaction conditions: *P* = 1 bar, *T* = 400 °C, H_2_: CO_2_ = 3:1; total flow rate = 18 mL·min^–1^.

The possible reaction intermediates and spectator species during CO_2_ hydrogenation were thoroughly investigated through a series of in situ DRIFTS experiments. Figure 4 shows the in situ time-resolved DRIFTS spectra of the RWGS reaction under near reaction conditions over CeO_2_ and FeCuCeO_2_ catalysts. Both spectra exhibit prominent broad bands at 1600 and 1270 cm^–1^, primarily arising from the symmetric and asymmetric stretching vibration modes of the bidentate carbonate (b-CO_3_^2–^) species.^37,38^ The broadening of these bands indicates the heterogeneous nature of the catalysts’ surfaces. To rule out the involvement of bicarbonate species in the evolution of these two bands, as they are expected to appear in the same spectral range, an experiment was conducted using deuterium (D_2_) instead of hydrogen (H_2_) in the reaction mixture. Interestingly, no red shift in the band positions was observed (Figure S8), suggesting that they are most likely related to bidentate carbonates. Along with b-CO_3_^2–^ species, additional weak shoulders at 1790, 1690, 1530, and 1410 cm^–1^, might be attributed to the formation of bridged carbonate (1790 cm^–1^),^39−41^ carboxylate (1690 cm^–1^),^42^ and monodentate carbonate (1530–1410 cm^–1^) are also observed.^42,43^ The triplet bands around 1000 cm^–1^ correspond to the C–O stretching modes of various carbonate species.^25,38^ Interestingly, the absence of asymmetric and symmetric OCO vibrations, as well as those characteristic of CH bonds in the 2850–2700 range of formate species, previously reported as an active intermediate for CO over CuCeO_2_,^3,4,41^ suggests that these species did not form to any significant extent in all of the catalysts under investigation. As illustrated in Figure 4, there is no appreciable difference in the nature of adsorbed species over pure CeO_2_ and Fe/CuCeO_2_, except for the amount of formed CO represented by the band at 2140 cm^–1^, which aligns well with catalytic activity. Identical in situ DRIFTS spectra were also observed on FeCeO_2_ and CuCeO_2_ catalysts (not shown), where bidentate carbonate species (b-CO_3_^2–^) predominated, along with other species. Similar DRIFTS experiments were performed at 300 and 350 °C (not shown), which show identical spectral features. This suggests that the low amounts of Fe and Cu did not play a role in tailoring the nature of adsorbed species, and all of these species are formed on the surface of the CeO_2_ support.

**Figure 5:**
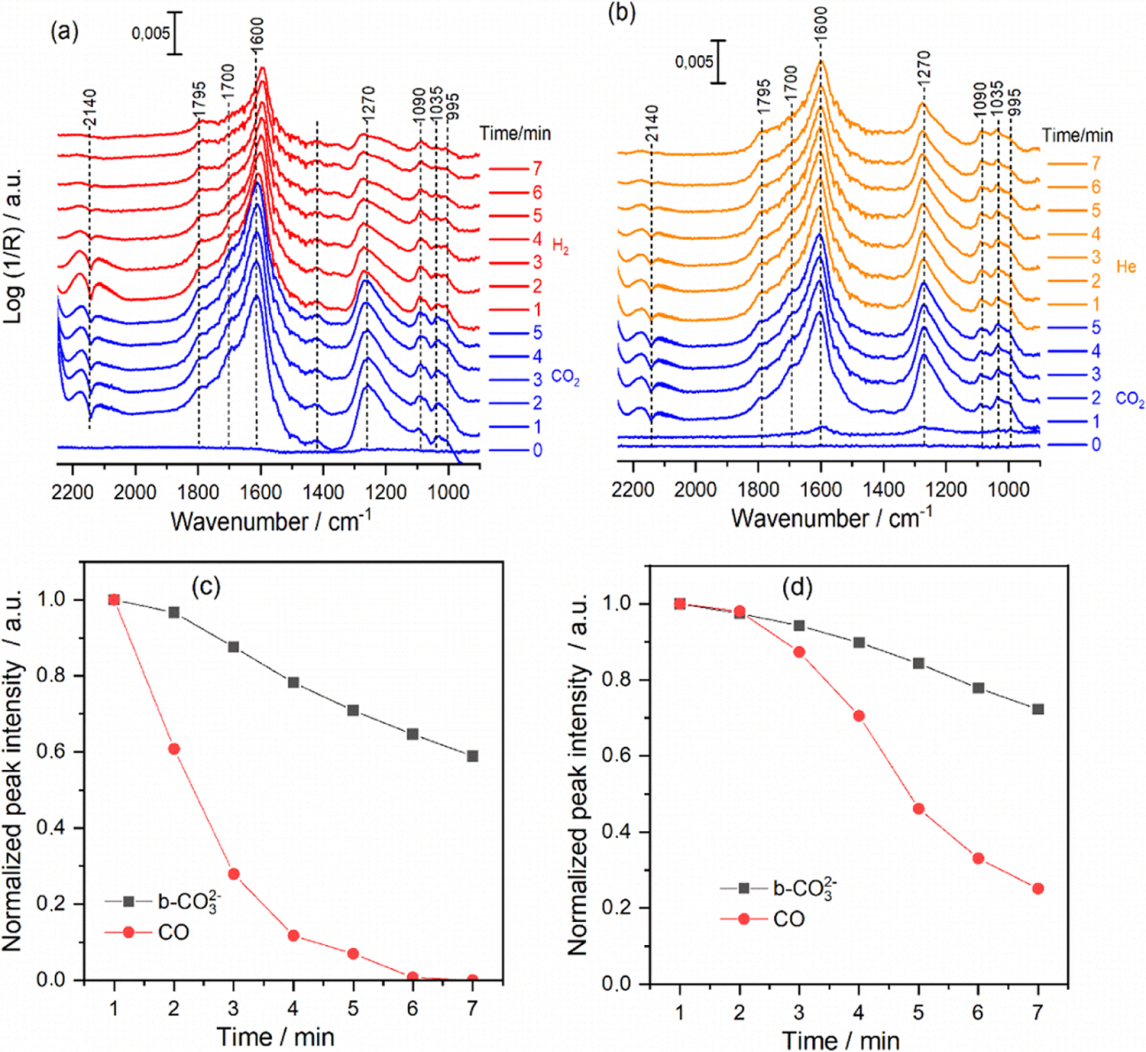
In situ time-resolved DRIFTS spectra obtained over Fe/CuCeO_2_ when the feed gas was switched between (a) 16.6% CO_2_ and 50% H_2_ (both in He), and (b) 16.6% CO_2_ (in He) and 100% He, and the corresponding evolution of normalized peak area/intensity of carbonate species (1600 cm^–1^) and gaseous CO (2140 cm^–1^) under a flow of (c) 50% H_2_ (in He) and (d) 100% He at 400 °C under *P* = 1 bar and a total flow rate of 18 mL·min^–1^.

To identify the species that predominantly contribute to the reaction, stepwise experiments were conducted by switching the reaction gas mixture from a CO_2_ + He atmosphere to a H_2_ + He atmosphere after in situ reduction at 400 °C under a flow of 50% H_2_ in He. The results are shown in Figure 5. It is noteworthy that following reduction, the catalysts underwent extensive purging under a flow of He (18 mL·min^–1^) at 400 °C for 6 h. Interestingly, as depicted in Figure 5a, after introducing a H_2_-free mixture of CO_2_ + He (blue spectra), a considerable amount of gaseous CO is formed. This observation may suggest that the dissociative adsorption of CO_2_ over the oxygen vacancies plays a crucial role in CO formation. Upon switching to H_2_, the band intensity of gaseous CO notably increased (red spectra after 1 and 2 min) and then significantly decreased. A similar stepwise experiment was also performed in with He (Figure 5b, orange spectra). In contrast, no increase in the band intensity of gaseous CO was observed. Figure 5 shows that b-CO_3_^2–^ species are the main adsorbed species formed during RWGS, and to determine if these species are active intermediates for CO formation, Figure 5c,d was plotted to show the corresponding changes in normalized peak intensity/area of b-CO_3_^2–^ species and gaseous CO as a function of time under the flow of both H_2_ and He. It was shown that the decay of b-CO_3_^2–^ is slightly faster under a H_2_ flow, as clearly shown in Figure S9. Moreover, it is clearly obvious from Figure 5c that at the time (≥6) when gaseous CO is no longer present in the gas phase, a considerable amount of b-CO_3_^2–^ still persists on the surface. A similar behavior was also noted for carboxylate and bridged carbonate species (Figure S10). This observation implies that these species do not serve as active intermediates in the formation of CO.

**Figure 6:**
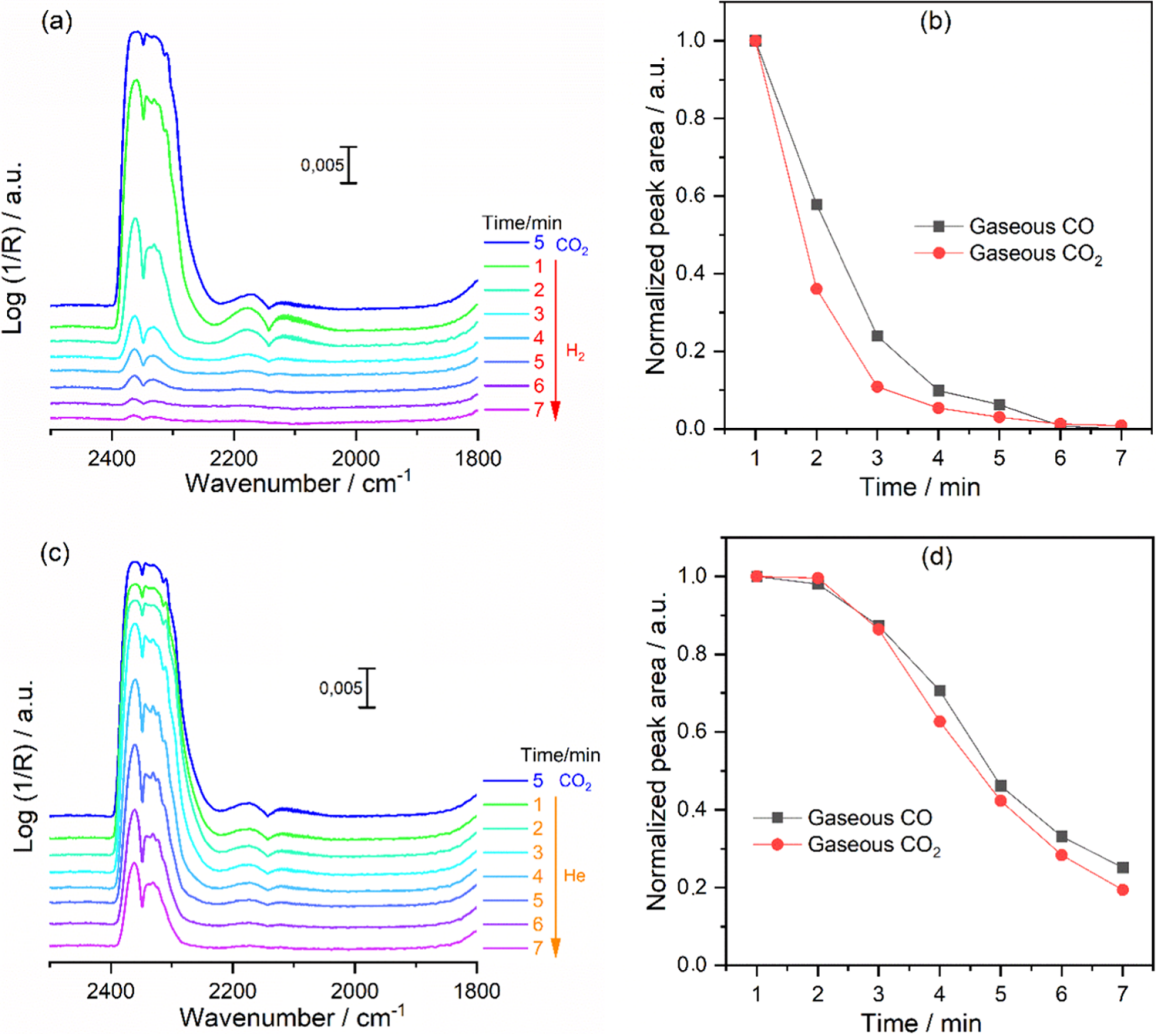
In situ time-resolved DRIFTS spectra over Fe/CuCeO_2_ showing the variation in the peak areas of gaseous CO_2_ and CO obtained under a flow of (a, b) 50% H_2_ (in He) and (c, d) 100% He, following the catalyst being exposed to 16.6% CO_2_ (in He) and reaching steady state (blue spectrum). Conditions: 400 °C, *P* = 1 bar, and total flow rate = 18 mL·min^–1^.

Figure 6 demonstrates a noteworthy alignment in the decay of gaseous CO_2_ band intensity alongside that of CO, under H_2_ and He flows, with a faster decay observed under a H_2_ flow. However, one may question and associate the rapid decrease in CO_2_ band intensity under a H_2_ flow with the efficient removal of CO_2_ from inside the cell, rather than being solely related to the RWGS reaction. To address this inquiry, we conducted an additional control experiment over SiO_2_, an inactive catalyst toward the RWGS reaction. It is evident from Figure S11a that although the rate of CO_2_ removal from inside the cell is higher under a H_2_ flow compared to He, it is still significantly slower than the rapid decay observed over Fe/CuCeO_2_ (Figure S11b). This suggests that the fast decay of CO_2_ shown in Figure 6b is primarily due to the RWGS reaction rather than the discharge of CO_2_ from the DRIFTS cell. The same scenario should be considered for the CO gas phase, as both gases are exposed to identical flow conditions.

Hence, the observed alignment in Figure 6b,d suggests a potential relationship, indicating that CO_2_ may undergo fast conversion into CO through an associative mechanism involving the formation of highly reactive adsorbed intermediate species. Alternatively, it could directly convert into CO through redox processes. It is important to note, however, that in both cases, we would expect to see a decrease in the band intensity of CO_2_ and a simultaneous increase in the band intensity of CO. However, operating under continuous flow conditions poses a challenge as the constant removal of CO from inside the cell prevents the anticipated rise in the band intensity of CO. Since the formation of CO through an associative mechanism via carboxylate, carbonate for formate is excluded, we suggest that CO could be primarily formed directly from the CO_2_ gas phase through a redox mechanism. However, to firmly propose that the redox mechanism is indeed the primary reaction pathway, it is essential to substantiate the simultaneous oxidation of the surface by CO_2_, an aspect that will be discussed in the subsequent sections.

Returning to Figure S9, a slightly more decrease in the b-CO_3_^2–^ band intensity is observed under a H_2_ flow compared to a He flow. As discussed, this decrease is not related to CO formation but is likely due to the thermal desorption of these species. Fundamentally, these species may be in a semidynamic equilibrium with gaseous CO_2_, indicating that b-CO_3_^2–^ forms on the surface as long as there is sufficient CO_2_ in the gas phase. Conversely, removal of CO_2_ from the gas phase leads to decomposition/desorption of CO_3_^2–^ as CO_2_. The lower amount of gaseous CO_2_ under a H_2_ flow, as shown in Figure 6a, compared to a He flow (Figure 6b), impacts the dynamic adsorption/desorption of carbonates, promoting thermal desorption under H_2_ flow conditions. This hypothesis is supported by Figure S12, where the decomposition/desorption of b-CO_3_^2–^ coincides with the nearly depleted CO_2_ gas phase within the cell.

**Figure 7:**
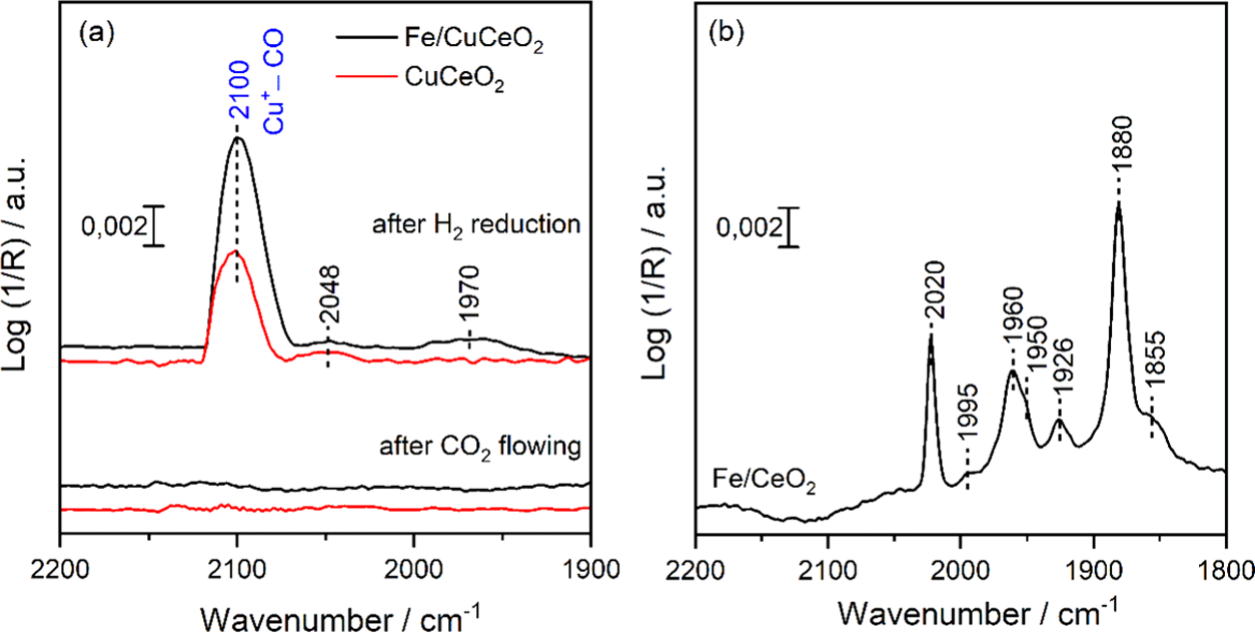
In situ DRIFTS spectra of CO adsorbed at room temperature on: (a) CuCeO_2_ and Fe/CuCeO_2_ after reduction at 400 °C for 2 h under 50%H_2_/He and a subsequent CO_2_ flow at 400 °C; (b) Fe/CeO_2_ after reduction at 400 °C for 2 h under 50%H_2_/He. All spectra were recorded after purging with He for 10 min.

The oxidation state and size of Cu and Fe in the catalysts were further explored through DRIFTS analysis of adsorbed CO. For Cu and Fe-based catalysts, it is widely recognized that lower wavenumber carbonyl bands are attributed to carbonyl species on metallic particles, while higher wavenumber bands indicate carbonyls on oxidized metal sites, with the wavenumber increasing with the metal oxidation state. Changes in carbonyl frequency are linked to variations in exposed faces and coordination of metal centers.^7,44−56^Figure 7a shows DRIFTS spectra for CO adsorption on both the fresh CuCeO_2_ and Fe/CuCeO_2_ catalysts after reduction and exposure to a CO_2_ flow at 400 °C. The spectra, recorded at room temperature following CO adsorption and subsequent purge in helium, clearly show the binding of CO to Cu^+^, as evidenced by the appearance of an IR band at 2100 cm^–1^ over both catalysts.^7,44,54,55^ The band intensity was higher over Fe/CuCeO_2_, indicating a higher density of Cu^+^ sites. Additionally, a very weak band at 2048 cm^–1^ is observed, likely due to CO adsorbed on Cu with higher metallic properties. For Fe/CuCeO_2_, an ill-defined weak band at 1970 cm^–1^ is present. Interestingly, after flowing CO_2_ for 15 min at 400 °C, the IR band at 2100 cm^–1^ disappeared, suggesting the reoxidation of Cu^+^ on the surface to Cu^2+^ by CO_2_. CO adsorption on Fe/CeO_2_ (Figure 7b) is also performed to study the behavior of Fe sites in comparison to Fe/CuCeO_2_. Surprisingly, the DRIFTS spectrum shows several bands at 2020, 1995, 1960, 1950, 1926, 1880, and 1855 cm^–1^. In the literature, there is a consensus that the CO adsorption bands in the 2050–1995 cm^–1^ range indicate the presence of linearly adsorbed CO on Fe^0^ sites,^49−53^ while bands spanning 1970–1880 cm^–1^ suggest the occurrence of bridge-bonded CO.^50,51^ Therefore, we attribute these bands in the range 2020–1995 cm^–1^ and 1960–1855 cm^–1^ to linearly and bridged adsorbed CO, respectively, on different types of Fe^0^ within Fe clusters. The absence of similar bands on Fe/CuCeO_2_ (Figure 7a, black lines), except for an ill-defined band at 1970 cm^–1^, suggests that the Fe species are highly dispersed and strongly interacting with CuCeO_2_. In contrast, Fe on CeO_2_ tends to segregate into small metallic Fe clusters during the reduction step.

#### 3.3.2 Operando EPR Studies

**Figure 8:**
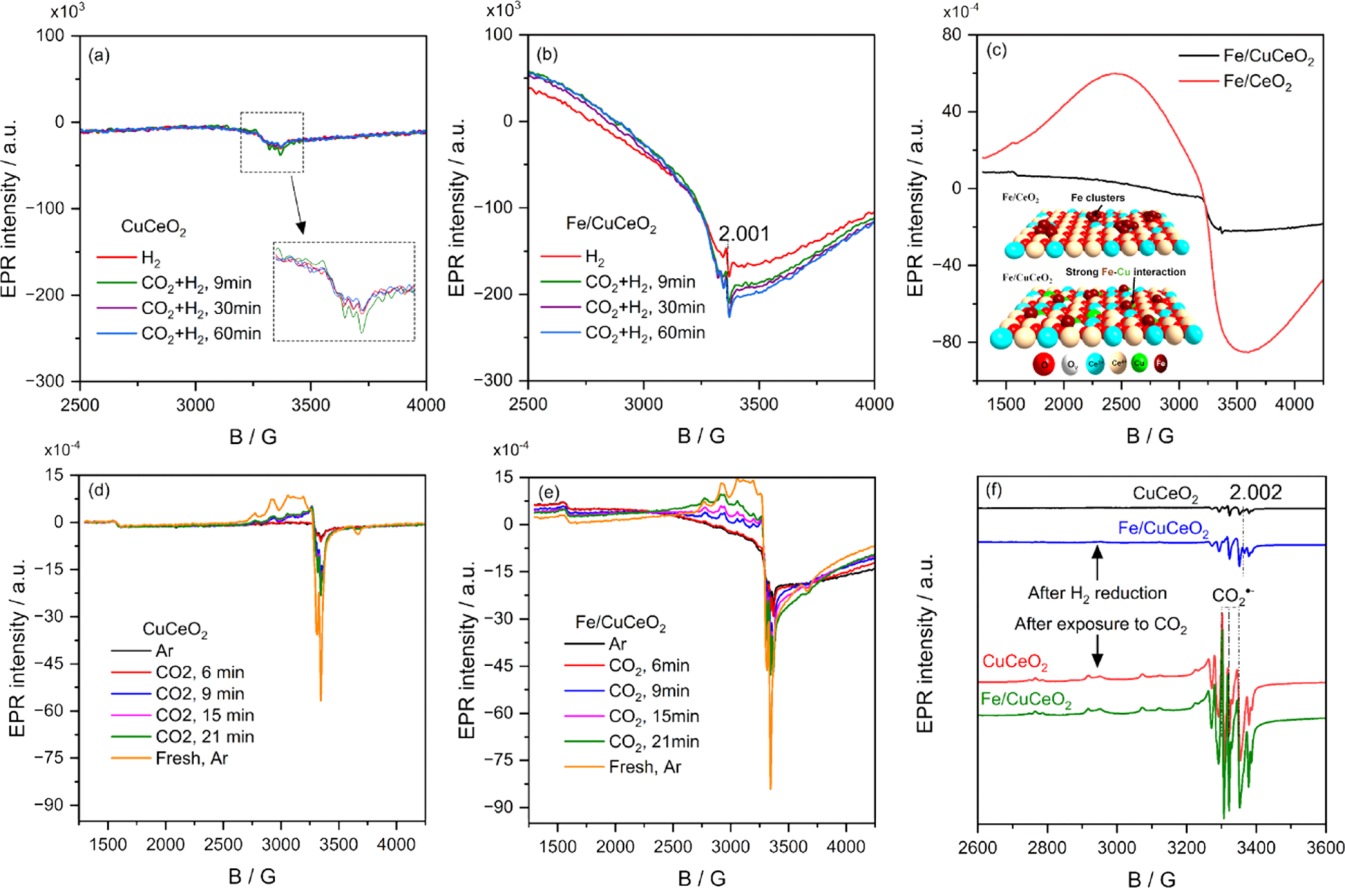
Operando EPR spectra of (a) CuCeO_2_ and (b) Fe/CuCeO_2_ recorded at 300 °C after reduction with 100% H_2_ (red line) and exposure to CO_2_ + H_2_/Ar (ratio CO_2_:H_2_ of 1:3). (c) In situ EPR spectra at 300 °C of ambient air-exposed fresh Fe/CeO_2_ and Fe/CuCeO_2_ catalysts after a 45 min pretreatment with a 50% H_2_/Ar flow with the inset showing a schematic illustration of the reduced Fe/CeO_2_ and Fe/CuCeO_2_ catalysts. Operando EPR spectra of (d) CuCeO_2_ and (e) Fe/CuCeO_2_ recorded at 300 °C after pretreatment with a 50% H_2_/Ar flow and flushing with Ar (black line) and then to a flow of 100% CO_2_ for 21 min. (f) Operando EPR spectra of CuCeO_2_ and Fe/CuCeO_2_ catalysts recorded at −180 °C after reduction (black and blue lines) and reoxidation with 100% CO_2_ (red and green lines).

Since operando DRIFTS does not provide direct evidence of the role of Cu^2+^, in situ/operando EPR investigations were conducted to monitor the behavior of Cu^2+^ under different gas conditions. As the CeO_2_ support and the Fe/CeO_2_ catalyst are inactive for RWGS at 300 °C, we will only focus on the results of operando EPR experiments at 300 °C for the two catalysts, CuCeO_2_ and Fe/CuCeO_2_ (Figure 8). We observe that for the less-active CuCeO_2_ catalyst, the Cu^2+^ signal slightly increases in the first 9 min (Figure 8a) revealing the oxidation of the initial EPR-silent Cu sites (Cu^+^) to Cu^2+^ species in the pre-reduced catalyst by CO_2_ under RWGS feed (compare red and green lines in Figure 8a). In subsequent redox cycles, these Cu^2+^ sites are reduced by H_2_ and reach a stable state after 30 min. In contrast, for the highly active Fe/CuCeO_2_ catalyst (Figure 8b), the EPR signals of both Cu^2+^ and O vacancy (*g* = 2.001) remain nearly unchanged after 9 min, indicating that a rapid dynamic oxidation/reduction of the redox active species by CO_2_ and H_2_, respectively, is established under RWGS conditions. The CO formation during operando EPR experiments was detected by online MS (Figure S13), indicating that the catalysts are active during the operando investigations.

To gain deep insights into the redox behavior of both catalysts, we conducted control experiments in which we followed the reduction and oxidation steps by H_2_ and CO_2_ flows, respectively. Figure S14 compares the reduction behavior of the CuCeO_2_ and Fe/CuCeO_2_ catalysts with that of the Fe/CeO_2_ catalyst. EPR spectra of CuCeO_2_ and Fe/CuCeO_2_ at 300 °C under an Ar flow revealed the coexistence of different Cu single sites with distinct coordination environments, accompanied by the formation of a small proportion of Cu dimers. For a detailed analysis of these Cu single sites and dimer species, refer to the EPR spectra at 25 °C for the air-exposed fresh catalysts in Figures S15 and S16, along with corresponding discussions in the Supporting Information file. Returning to the reduction behavior of CuCeO_2_ and Fe/CuCeO_2_ catalysts, it is evident from Figure S14a,b that the EPR signal of both Cu^2+^ monomers and dimers almost vanished rapidly due to the reduction of Cu^2+^ to EPR-silent Cu^+^ species. Additionally, the EPR spectra of Fe/CuCeO_2_ exhibited a broad signal which increased with prolonged reduction time, suggesting the formation of magnetically interacting Fe species (Figure S14b). In contrast to CuCeO_2_, the clear appearance of O-vacancy signals at g = 2.002 indicates a higher amount of O vacancy in this catalyst. Upon comparing the EPR spectra of both Fe/CeO_2_ (Figure S14c) and Fe/CuCeO_2_ catalysts after a 45 min reduction, it is evident that the broadening of the signal of the magnetically interacting Fe species is significantly more pronounced in Fe/CeO_2_ compared to Fe/CuCeO_2_ (Figure 8c). This can be explained by the highly dispersed nature of Fe on CuCeO_2_ due to the strong interaction between Fe and Cu species. This strongly supports the idea that a significant portion of Fe preferably interacts with Cu, as suggested by H_2_-TPR. In contrast, on pure CeO_2_, Fe tends to agglomerate, in line with the formation of Fe clusters after reduction, as evidenced by DRIFTS CO adsorption.

When the reduced CuCeO_2_ and Fe/CuCeO_2_ catalysts are purged with Ar and subsequently exposed to a CO_2_ flow, the intensities of the EPR signals of Cu^2+^ ions remarkably increase again due to the reoxidation of the EPR-silent Cu^+^ species (Figure 8d,8e). For CuCeO_2_ (Figure 8d), the intensity of EPR signals of Cu^2+^ ions after 21 min is still far from those of the fresh catalyst (orange line, Figure 8d), suggesting the presence of EPR-silent species (Cu^+^) that cannot be easily oxidized by CO_2_. This effect is clearly observed in Figure S18, where the EPR signal double integrals show a very slow increase in Cu^2+^ signals after 9 min. In contrast, for the more active Fe/CuCeO_2_ catalyst (Figure 8e), the EPR signal continues to increase and does not reach a stable state even after 21 min, approaching the intensity of the fresh catalyst (orange line). After 21 min under a CO_2_ flow, the Fe/CuCeO_2_ catalyst exhibited a 3.4 times higher relative amount of Cu^2+^ sites than CuCeO_2_, despite both catalysts having a similar Cu content, as evident from ICP measurements, as they were prepared from the same CuCeO_2_ batch. These results emphasize the significant role of Fe in promoting the reoxidation step of Cu^+^ by CO_2_. Our findings contradict an earlier study by Chen et al.,^19^ which proposed that the addition of Fe inhibits the oxidation of Cu.

After reduction in H_2_ at 300 °C and subsequent reoxidation by CO_2_ for 21 min, the catalysts were first cooled down to RT under a continuous flow of CO_2_ and then purged with Ar to remove gaseous CO_2_, before collecting the EPR spectra at −180 °C (Figure 8f). The spectra revealed a pronounced orthorhombic signal (*g*_*xx*_ = 2.0048, *g*_*yy*_ = 2.0275, *g*_*zz*_ = 2.0039), indicating the presence of adsorbed CO_2_^•–^ radicals, along with the distinct Cu^2+^ features. It is important to note that these CO_2_^•–^ radicals can only be detected when the catalyst is cooled to RT under a continuous flow of CO_2_. When purged with Ar at 300 °C, these radicals are easily removed, demonstrating their high instability. These radicals, previously observed in various Cu/CeO_2_ catalysts and on carbonate surfaces in synthetic apatite,^57−59^ likely result from CO_2_ adsorption on oxygen vacancies. In this process, the Ce^3+^ formed during the reduction step donates an electron to adsorbed CO_2_, forming a Ce^4+^–CO_2_^•–^ moiety. Further confirmation was achieved using DMPO as a spin trap, revealing the interaction between the CO_2_^•–^ radical generated upon CO_2_ adsorption on reduced Fe/CuCeO_2_ and DMPO, forming a DMPO-CO_3_^•^ adduct (*g* = 2.0069, *A*_N_ = 12.90 G, *A*_H_^β^ = 6.48 G, *A*_H_^γ^ = 1.88 G), as depicted in Figure S19.

#### 3.3.3 NAP-XPS Measurement

**Figure 9:**
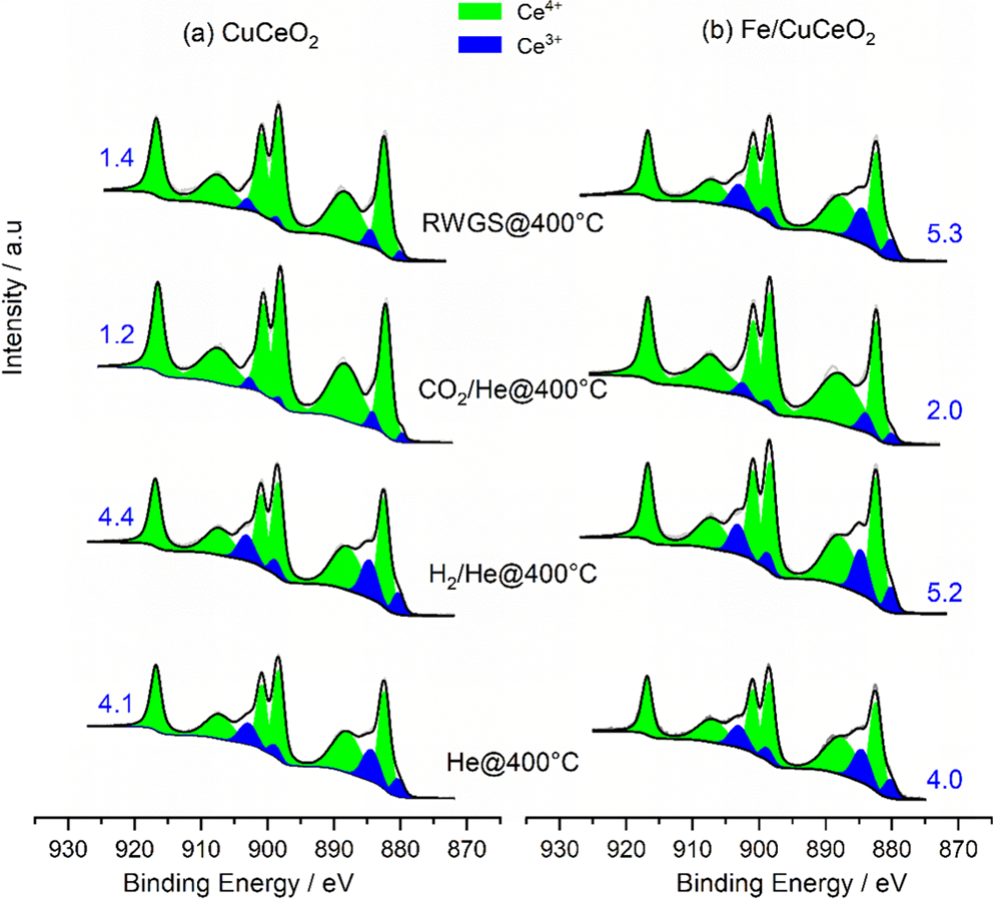
High-resolution XPS spectra of the Ce 3d region recorded during NAP-XPS experiments at the indicated conditions for (a) CuCeO_2_ and (b) Fe/CuCeO_2_ catalysts. Blue numbers represent the surface content (%) of Ce^3+^.

To investigate the role of Ce^3+^ in the RWGS reaction, the interaction of CO_2_ and CO_2_/H_2_ mixture with CuCeO_2_ and Fe/CuCeO_2_ surfaces was examined using NAP-XPS. Figure 9 shows a series of XP spectra collected under different conditions. Special attention was given to the Ce 3d and C 1s regions, as the low loadings of both Cu and Fe prevent the direct observation of these elements in NAP-XPS experiments. The Ce 3d XPS region exhibits distinct and complex features, encompassing 10 components designated according to the notation introduced by Burroughs et al.^60^ Specifically, the peaks corresponding to Ce 3d_5/2_ are identified as *v*_0_, *v*, *v*′, *v*″, and *v*‴, while those corresponding to Ce 3d_3/2_ are labeled *u*_0_, *u*, *u*′, *u*″, and *u*‴. Furthermore, the spin–orbit split doublets *v*_0_ = 880.6 eV, *u*_0_ = 899.2 eV, *v*′ = 884.8 eV, and *u*′ = 903.6 eV are associated with the Ce^3+^ components, whereas the remaining peaks (*v* = 882.5 eV, *v*″ = 887.6 eV, *v*‴ = 898.3 eV, *u* = 901.1 eV, *u*″ = 907.7 eV, *u*‴ = 917 eV) are attributed to the Ce^4+^ components. The surface percentage of Ce^3+^ (indicated by the blue numbers on the right and left sides of the spectra in Figure 9) was determined from the area of the subpeaks using eq 4:
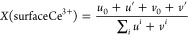
4

For the CuCeO_2_ catalyst, both the fresh catalyst under a He flow at 400 °C and the reduced form after in situ reduction in 50%H_2_/He at 400 °C show similar surface Ce^3+^ content (4.1–4.4%). The resemblance in Ce^3+^ content suggests that heating the catalyst at 400 °C under a He flow may be sufficient for the reduction of surface CeO_2_ and, consequently, for the removal of most loosely bound surface oxygen. Exposure of the reduced catalyst to CO_2_ at 400 °C led to a significant decrease in the amount of surface Ce^3+^ (1.2%), suggesting the reoxidation of Ce^3+^ into Ce^4+^. The oxidation of Ce^3+^ to Ce^4+^ under these conditions could be attributed to the adsorption and dissociation of CO_2_ on oxygen vacancies, leading to CO formation and filling the oxygen vacancies. This is consistent with the EPR spin-trap experiment, which suggests electron transfer from oxygen vacancies to CO_2_ (Figure S19). However, under the flow of the RWGS mixture, the amount of Ce^3+^ increased slightly. Whereas fresh Fe/CuCeO_2_ contains a comparable amount of Ce^3+^ to CuCeO_2_, while the reduced form contains a relatively higher amount, suggesting the facile reduction of the catalyst in the presence of Fe, consistent with H_2_-TPR results. Similar to CuCeO_2_, a significant decrease in the amount of Ce^3+^ occurred after exposing the catalyst to CO_2_ due to the oxidation of Ce^3+^ via the dissociative adsorption of CO_2_. Interestingly, when Fe/CuCeO_2_ was exposed to the RWGS gas mixture, the amount of Ce^3+^ returned to its value on the reduced form. These findings suggest that in the presence of Fe, the reduction and oxidation steps of Ce^4+^/Ce^3+^ cycles are very fast under steady-state conditions.

In contrast to Fe/CuCeO_2_, the observed difference in the percentage of Ce^3+^ over CuCeO_2_ after reduction compared to that during the RWGS reaction (4.4% versus 1.2%) indicates that some Ce^4+^ ions formed under RWGS conditions cannot be easily reduced back to Ce^3+^. This suggests that the oxidation step (Ce^3+^ → Ce^4+^) is more effective than their reduction (Ce^4+^ → Ce^3+^). However, the EPR findings emphasize that the oxidation of Cu^+^ in Cu^+^–O_v_–Ce^3+^ moieties into Cu^2+^ on this catalyst is less efficient compared to Fe/CuCeO_2_. This clearly indicates that the coexisting Ce^4+^ with Cu^2+^ in the same Cu^2+^–O^2–^–Ce^4+^/Cu^+^–O_v_–Ce^3+^ moieties are not responsible for this discrepancy, as they should exhibit similar redox behavior to their Cu^2+^/Cu^+^ counterparts. Therefore, other Ce^4+^-containing sites, most probably CeO_*x*_ domains (Ce^4+^–O^2–^–Ce^4+^), are involved in the reaction and responsible for this observed discrepancy. These sites become highly reducible after the addition of Fe, aligning well with the H_2_-TPR results that indicate the formation of additional Fe^3+^–O^2–^–Ce^4+^ sites with highly reducible properties on Fe/CuCeO_2_, at the expense of CeO_*x*_ domains on CuCeO_2_ (Figure 1d).

Figure S20 shows C 1s signals obtained during the experiments. For reduced CeO_2_, distinct signals at 284.8 and 289.4 eV, attributed to adventitious carbon and carbonate species, are evident.^61^ Similar signals are observed for the reduced CuCeO_2_ and Fe/CuCeO_2_ catalysts. Compared with bare CeO_2_, following the reduction step, a slight decrease in the intensity of the signals at 284.8 and 289.4 eV is observed for CuCeO_2_, while a significant reduction is observed for Fe/CuCeO_2_. Upon exposing the reduced catalysts to a mixture of H_2_ and CO_2_ at 400 °C, a small peak at 293.3 eV related to gaseous CO_2_ appeared.^62^ Under a He flow, this signal disappears. The low intensity of the signal at 289.4 eV suggests that associative mechanisms play no role in CO formation, consistent with in situ DRIFTS conclusions.

### 3.4 Structure–Activity Relationship

In situ/operando EPR demonstrates that copper is predominantly dispersed as a single atom on CeO_2_ (Cu^2+^–O^2–^–Ce^4+^). H_2_-TPR results suggest that the high fraction of Fe prefers to strongly interact with Cu sites (Cu^2+^–O^2–^–Ce^4+^), while another fraction interacts with CeO_*x*_ forming highly dispersed and reducible Fe^3+^–O^2–^–Ce^4+^ sites. The combined results from operando EPR and in situ DRIFTS of CO adsorption reveal a significant difference in the behavior of Fe on CeO_2_ and CuCeO_2_. The added Fe is highly dispersed on CuCeO_2_ and resists agglomeration during the reduction step, while on CeO_2_, it rapidly agglomerates, forming Fe clusters. The robust interaction between Fe and Cu stabilizes a more proportion of single-atom Cu sites compared to the iron-free catalyst (CuCeO_2_). The addition of Fe facilitates the formation of a higher amount of surface oxygen vacancies as evidenced from the intense DMPO/O_2_^•–^ adduct observed by in situ EPR (Figure S17). This subsequently results in a relatively higher percentage of surface Ce^3+^, as evidenced by NAP-XPS and EELS spectra. The addition of Fe was observed to markedly enhance the reduction of CeO_*x*_ and the reoxidation of Cu^+^ sites on the Fe/CuCeO_2_ catalyst, as confirmed by NAP-XPS and operando EPR, respectively.

The in situ DRIFTS analysis emphasizes the predominant formation of b-CO_3_^2–^ on all catalysts, accompanied by smaller quantities of bridged and monodentate CO_3_^2–^ and carboxylate species. Detailed in situ DRIFTS switching experiments exclude the involvement of these species in CO formation through an associative mechanism. Instead, a notable correlation is observed between the decay in the band intensity of both gaseous CO_2_ and CO under both H_2_ and He flow conditions, suggesting the redox mechanism as the primary reaction pathway. The surface oxidation of Cu^+^, as confirmed by Operando EPR and in situ DRIFTS of CO adsorption, coupled with the simultaneous surface oxidation of Ce^3+^ revealed through NAP-XPS under CO_2_ flow, lends further support to the redox mechanism. The formation of CO^2•–^ radicals, resulting from electron transfer from oxygen vacancies, as demonstrated by in situ spin-trap EPR experiments, may play a pivotal role in facilitating the dissociative adsorption of CO_2_ into CO and O. This leads to the filling of oxygen vacancies and, consequently, surface oxidation. In conclusion, the redox pathway, along with the enhanced redox properties observed after the addition of Fe, can explain the superior activity of Fe/CuCeO_2_ compared to CuCeO_2_. This study suggests that the Cu^2+^–O^2–^–Ce^4+^/Cu^+^–O_v_–Ce^3+^ ensembles and the highly dispersed Fe^3+^–O^2–^–Ce^4+^ sites, with highly reducible (O^2–^) oxides, serve as the active sites for RWGS.

**Scheme 1:**
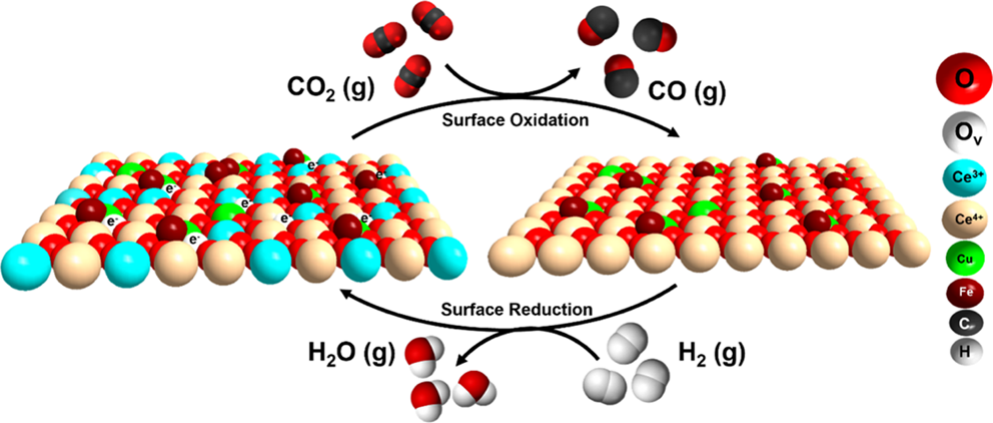
Overview of the Redox Mechanism and Surface Structure Changes on the Fe/CuCeO_2_ Catalyst during the RWGS Reaction

Based on the preceding discussion, we propose the redox mechanism depicted in Scheme 1 as the primary reaction pathway for both unmodified and iron-modified CeO_2_-supported Cu single-atom sites. In this redox cycle, hydrogen dissociates over Cu^2+^ sites with the help of nearby oxygens.^63^ It subsequently migrates and reduces the CeO_2_ surface, leading to the generation of oxygen vacancies. The subsequent step involves the dissociative adsorption of CO_2_ onto these oxygen vacancies, resulting in surface oxidation and the simultaneous release of CO. While the redox mechanism has been previously demonstrated by correlating steady-state RWGS activity with the number of active surface oxygen and its reduction kinetics,^64−66^ this study exploits the change in the peak area of IR bands of CO_2_ and CO gaseous phase coupled with the evidence of the surface oxidation of Ce^3+^ and Cu^+^ by CO_2_ from NAP-XPS, in situ DRIFTS of CO adsorption, and operando EPR as a straightforward approach for elucidating the redox pathway.

## 4 Conclusions

In this study, CeO_2_-supported single-atom Cu catalysts modified and unmodified with iron were prepared and tested for RWGS reaction. The catalytic results unveil a significant synergistic effect between CuCeO_2_ and Fe, indicating an activity three times higher than the combined catalytic activities of monometallic catalysts under identical conditions. Detailed in situ DRIFTS, operando EPR, and NAP-XPS experiments were applied to unravel the reaction mechanism and the role of Fe. The results imply the formation of two different active sites for the RWGS reaction on Fe/CuCeO_2_, consisting of Cu^2+^–O^2–^–Ce^4+^ ensembles and Fe^3+^–O^2–^–Ce^4+^ sites. The addition of iron (Fe) led to a significant enhancement in the reoxidation process of Cu^+^ to Cu^2+^ and the reduction of Ce^4+^ under the given reaction conditions. Neither in situ DRIFTS nor NAP-XPS detected the presence of surface formate species during RWGS, which is previously suggested as the active intermediate species. The combined results suggest that the RWGS reaction mechanism proceeds via the redox pathway. This involves the formation of CO_2_^•–^ radicals on oxygen vacancies, which eventually decompose to CO. Consequently, the findings from this study will play a crucial role in advancing the rational design of highly efficient and selective catalysts, through engineering catalysts with oxygen vacancy-rich surfaces, not only for RWGS but also for various other redox reactions.

## Supporting Information Available

Additional data on characterization, catalytic performance, and in situ/operando investigations (Figures S1–S20 and Tables S1–S3) (PDF)
