# Supplementary material for: CeO2-Supported Single-Atom Cu Catalysts Modified with Fe for RWGS Reaction: Deciphering the Role of Fe in the Reaction Mechanism by In Situ/Operando Spectroscopic Techniques
Source: ACS Catal. 2024 Jul 5;14(14):10913–27. doi: 10.1021/acscatal.4c01493 (PMC11265290; doi:10.1021/acscatal.4c01493)
Supplement: Supplementary file 1 — cs4c01493_si_001.pdf [file cs4c01493_si_001.pdf]

Supporting Information

CeO_2-Supported Single-Atom Cu Catalyst Modified with Fe for RWGS Reaction: Deciphering the Role of Fe in the Reaction Mechanism by In-Situ/Operando Spectroscopic Techniques

Abdallah I. M. Rabee,[a,b]* *Hayder Abed,*[a] *Thanh Huyen Vuong,*[a] *Stephan Bartling,a Laura Kraußer,*[a] *Hanan Atia,*[a] *Nils Rockstroh,*[a] *Evgenii V. Kondratenko,*[a] *Angelika Brückner,*[a,c] *Jabor Rabeah*[a,d]*

[a] Leibniz-Institut für Katalyse, Albert-Einstein-Str. 29A, 18059 Rostock, Germany

[b] Chemistry Department, Faculty of Science, Minia University, El-Minia, 61519, Egypt

[c] Department Life, Light and Matter, University of Rostock, Albert-Einstein-Str. 25, 18059 Rostock, Germany

[d] State Key Laboratory of Low Carbon Catalysis and Carbon Dioxide Utilization, Lanzhou Institute of Chemical Physics (LICP), Chinese Academy of Sciences, Lanzhou 730000, P. R. China

*Correspondence: Abdallah.Ahmed@catalysis.de and Jabor.Rabeah@catalysis.de

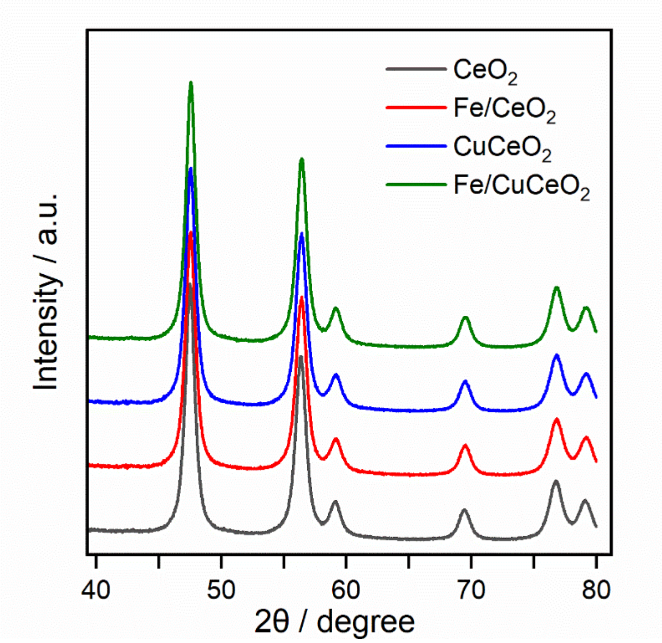

Figures S1. X-ray diffraction patterns of the indicated catalysts and the bare support.

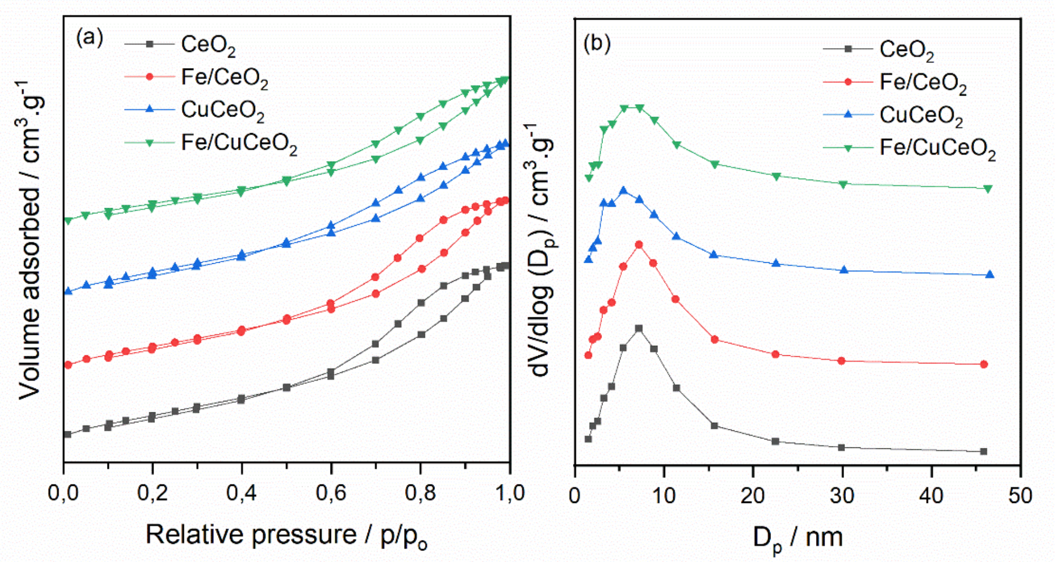

Figures S2. (a) N_2 adsorption-desorption isotherms and (b) pore size distribution of the indicated catalysts and the bare support.

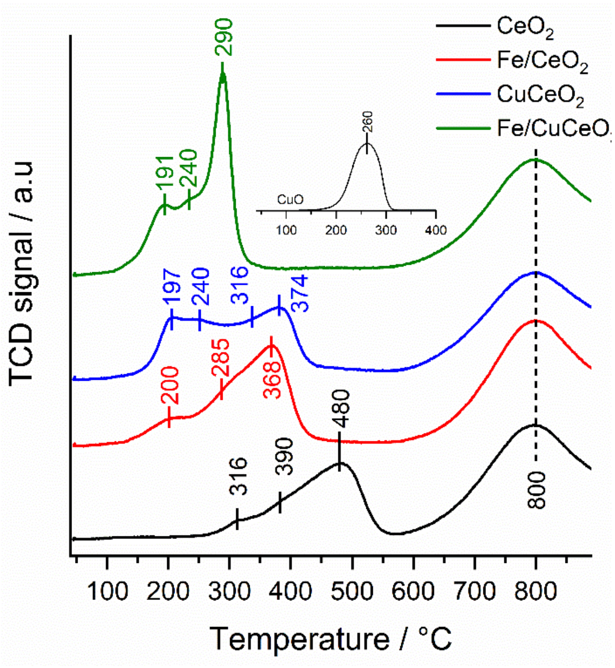

Figure S3. H_2-TPR profiles recorded as a function of temperature for the indicated catalysts. Inset is the TPR of CuO standard.

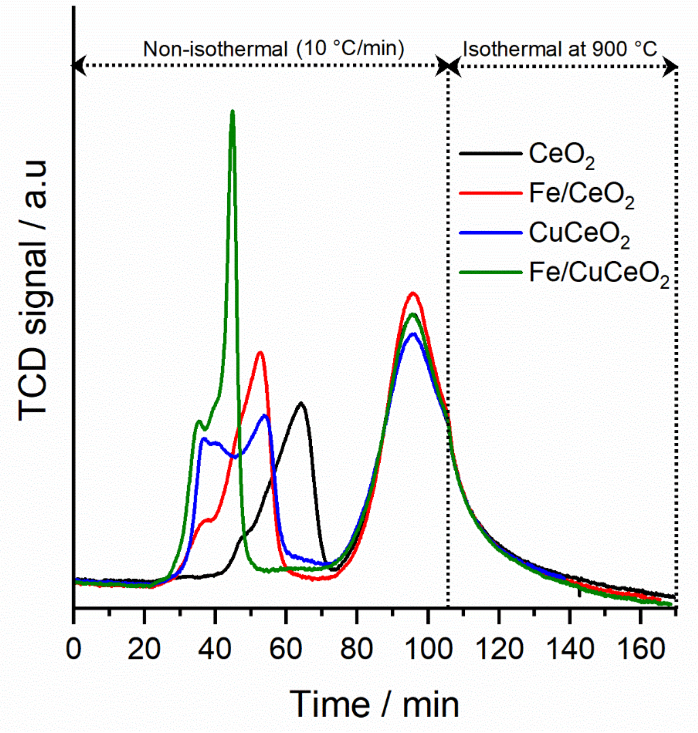

Figure S4. H_2-TPR profiles recorded as a function of time for the indicated catalysts. The TPR profiles were recorded via non-isothermal reduction up to 900°C, followed by an isothermal hold at 900°C until the completion of reduction, and the profile returned to its baseline.

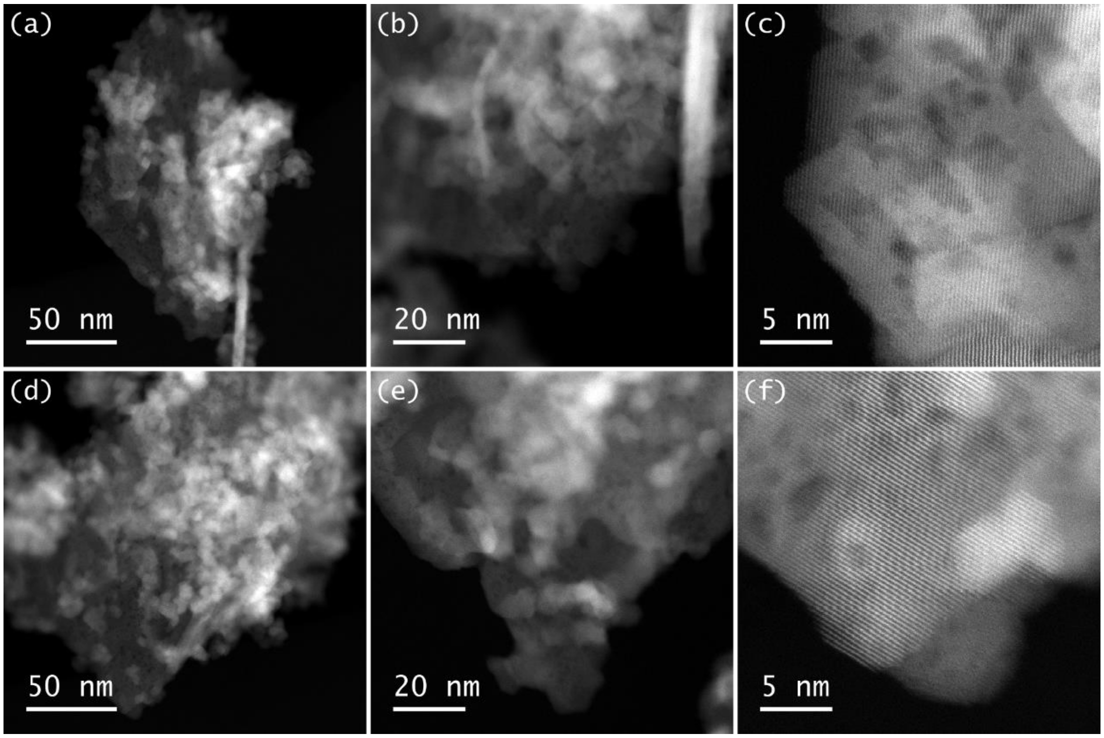

Figure S5. Selected HAADF-STEM images of (a, b, c) $CuCeO_2$ and (d, e, f) Fe/$CuCeO_2$ after *ex-situ* reduction at 400°C for 2 h under a flow (13 ml·min^{-1}) of 50%H_2 in Ar.

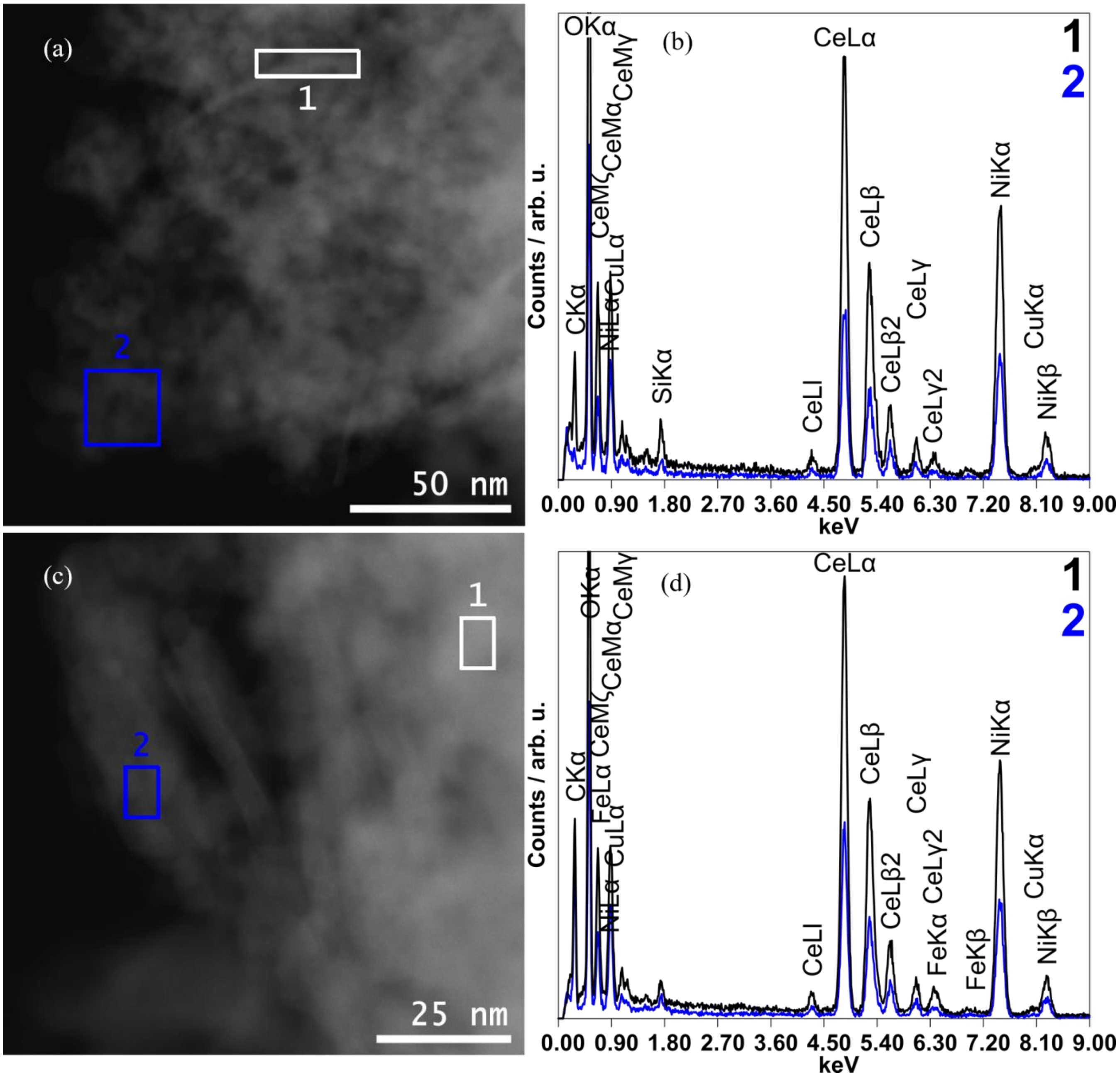

Figure S6. Scanning transmission electron microscopy – high-angle annular dark field (HAADF-STEM) images along with the corresponding EDX spectra obtained from distinct regions highlighted by rectangles in the HAADF images of (a,b) $CuCeO_2$ and (c,d) $Fe/CuCeO_2$ catalysts. Please note that the Cu K_α peak is slightly visible in each material (spectrum 1 in b and spectrum

1 in d), but that this signal is in the range of the limit of detection. Iron cannot be seen as this signal is superimposed by the signals of Ce.

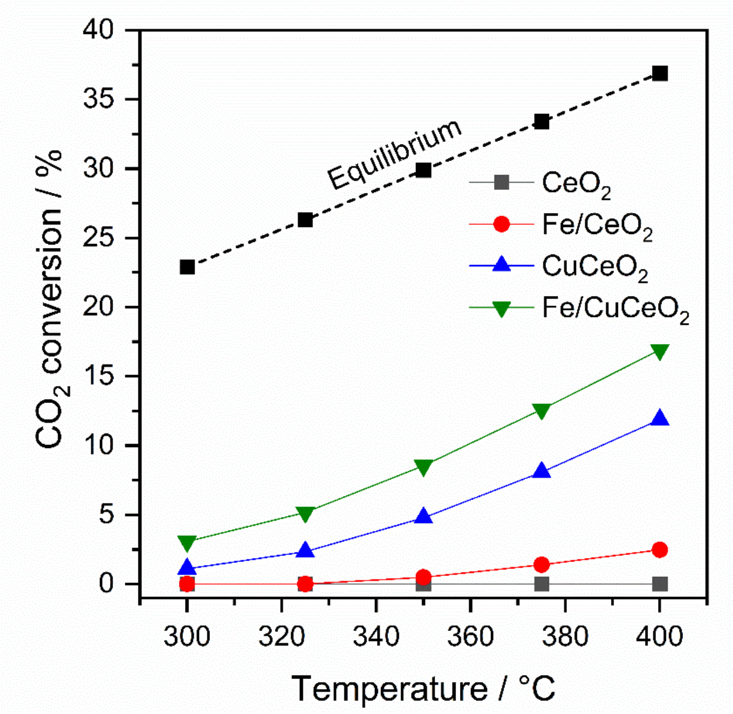

Figure S7. CO_2 conversion for RWGS reaction for CeO_2, Fe/CeO_2, CuCeO_2, and Fe/CuCeO_2 catalysts in the temperature range of 300-400 °C. Reaction condition: P = 1 atm, CO_2:H_2 = 1:3, GHSV = 36000 mL·g^{-1}·h^{-1}.

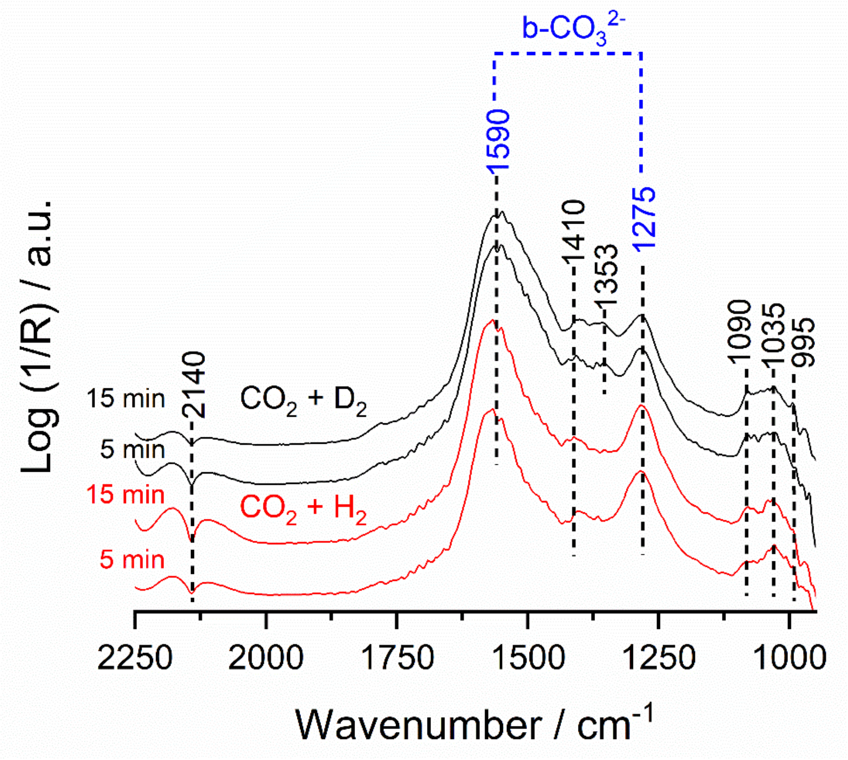

Figure S8. In-situ DRIFTS spectra of the RWGS reaction at 400°C over reduced Fe/CuCeO$_2$ catalyst under a flow of H_2:CO_2:He (3:1:2) (red spectra) before switching to D_2:CO_2:He (3:1:2) (black spectra) at 18 ml.min^{-1}. Reduction conditions: P = 1 bar, T = 400°C, 50%H_2/He, total flow rate = 13 ml.min^{-1}.

Figure S8 represents DRIFTS spectra obtained when the reduced catalyst was initially exposed to the RWGS reaction mixture (CO_2 + 3H_2 + 2He) for 15 minutes, followed by switching the gas flow to CO_2 + 3D_2 + 2He. This DRIFT experiment was performed to detect any potential red shift in the IR bands due to deuterium-hydrogen exchange and to determine if the bending vibration of bicarbonate species (δCOH) contributes to the appearance of the band at 1275 cm^{-1}. Remarkably, after H_2 was replaced with D_2 in the reaction mixture, the bands corresponding to the predominant species, characterized by the bands at 1590 cm^{-1} and 1275 cm^{-1}, remained at the same positions

(**Figure S8**), indicating that these bands likely represent the symmetric and asymmetric stretching vibration modes of bidentate carbonates.

However, it is important to mention that after switching to $CO_2 + D_2$, two additional observations were made. Firstly, in addition to the small band at 1410 cm^{-1}, which was assigned to monodentate carbonate, we observed another very small band at 1353 cm^{-1}. The origin of this band may be related to the formation of additional carbonate species resulting from the change in surface reducibility when H_2 is replaced with D_2 during the reaction. Secondly, the amount of evolved CO was slightly less compared to the reaction with H_2. This could be attributed to the reducing capability of D_2 in comparison with H_2.

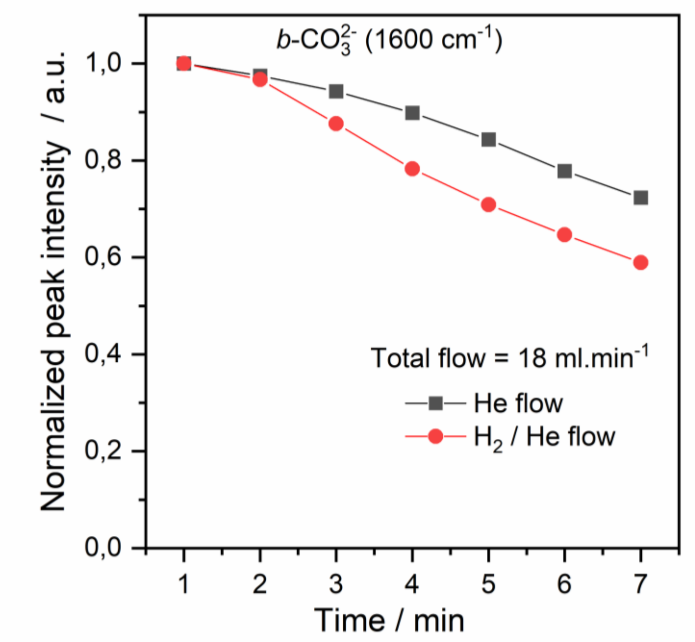

Figure S9: (a) Normalized peak intensity of bidentate carbonates ($b\text{-}CO_3^{2-}$) under a flow of 100% He and 50% H_2/He over Fe/$CuCeO_2$ catalyst. Conditions: 400 °C under P = 1 bar, and total flow rate = 18 $ml.min^{-1}$.

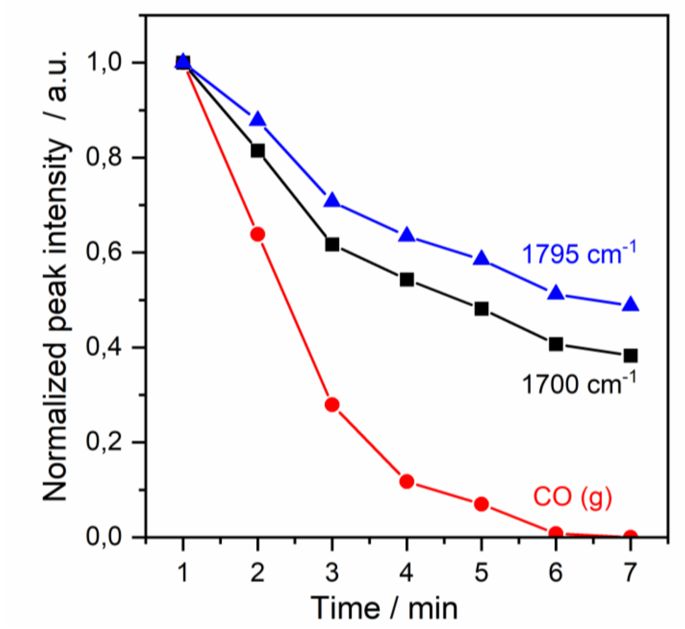

Figure S10: (a) Normalized peak area/intensity of gaseous CO, carboxylate (1700 cm^{-1}) and bridged carbonate (1795 cm^{-1}) under a flow of 50% H_2/He over Fe/CuCeO$_2$. Conditions: 400 °C under P = 1 bar, and total flow rate = 18 ml.min^{-1}.

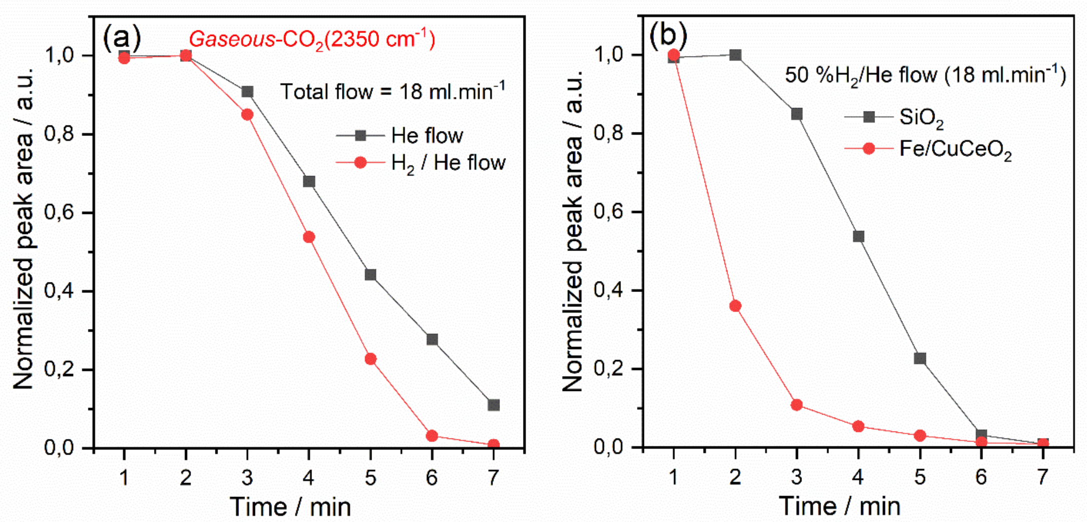

Figure S11: (a) Normalized peak area of gaseous CO_2 under a flow of 50% H_2/He and 100% He over SiO_2, and (b) a comparison of the normalized peak area of gaseous CO_2 under a flow of 50% H_2/He over Fe/CuCeO$_2$ and SiO_2. Conditions: 400 °C under P = 1 bar, and total flow rate = 18 ml.min^{-1}.

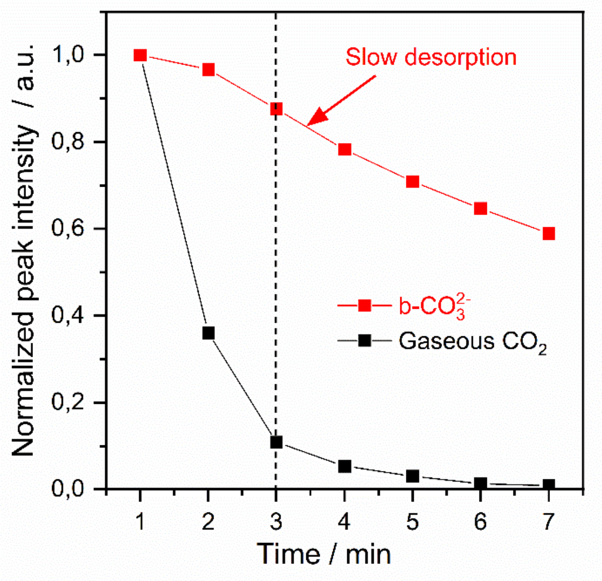

Figure S12: Normalized peak area of gaseous CO_2 and bidentate carbonate (1600 cm^{-1}) under a flow of 50% H_2/He over Fe/CuCeO_2. Conditions: 400 °C under P = 1 bar, and total flow rate = 18 $ml.min^{-1}$.

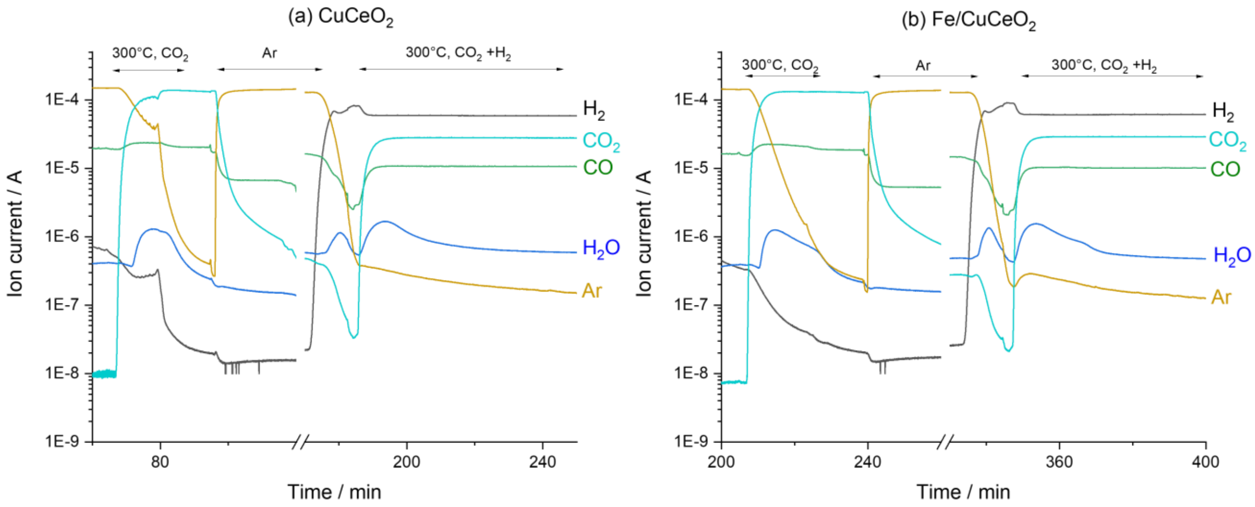

Figure S13. Mass spectrometric analysis of the effluent gas stream detects CO and H_2O formation over (a) CuCeO_2 and (b) Fe/CuCeO_2 catalysts at 300 °C, conducted in parallel with operando EPR measurements.

The MS signals of H_2, H_2O, CO, Ar, and CO_2 recorded parallel to the operando EPR spectra are shown in **Figure S13**. Upon exposure to only CO_2, the formation of CO slightly increased, while it increased significantly during the RWGS reaction (under CO_2+H_2/Ar flow, with a ratio of CO_2/H_2 of 1/3) on both catalysts. Comparing the amount of CO formation under CO_2 flow, the degree of increase in CO formation for the catalyst Fe/$CuCeO_2$ (40%) is higher than that for $CuCeO_2$ (27%), which aligns well with the activity results for these catalysts. Moreover, the higher amount of CO formed under CO_2 flow in the Fe/$CuCeO_2$ sample indicates its higher number of oxygen vacancies and higher oxygen mobility.

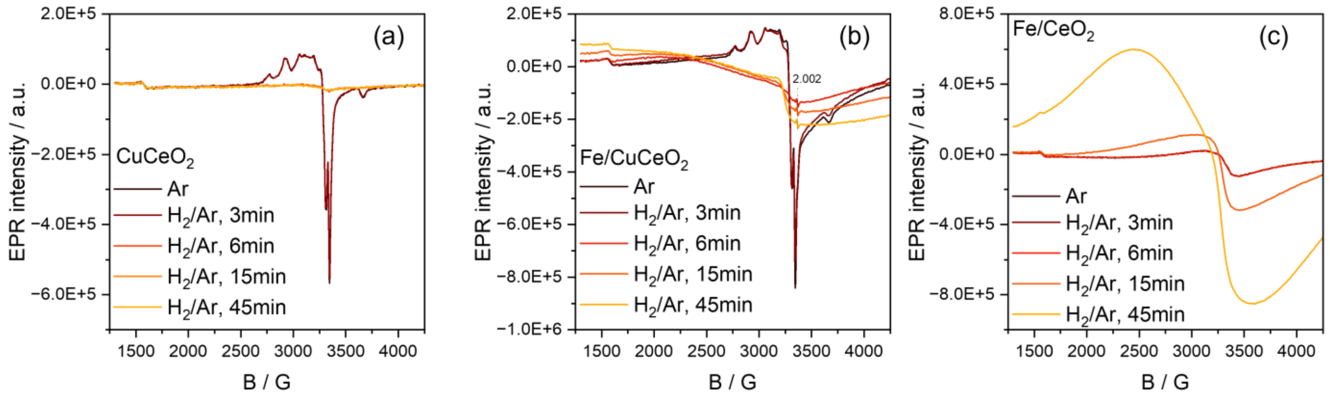

Figure S14. In-situ EPR spectra at 300 °C of ambient air-exposed fresh catalysts (black spectrum) and during a 45-minute pretreatment with 50% H_2/Ar flow for (a) $CuCeO_2$, (b) Fe/$CuCeO_2$, and (c) Fe/CeO_2 catalysts.

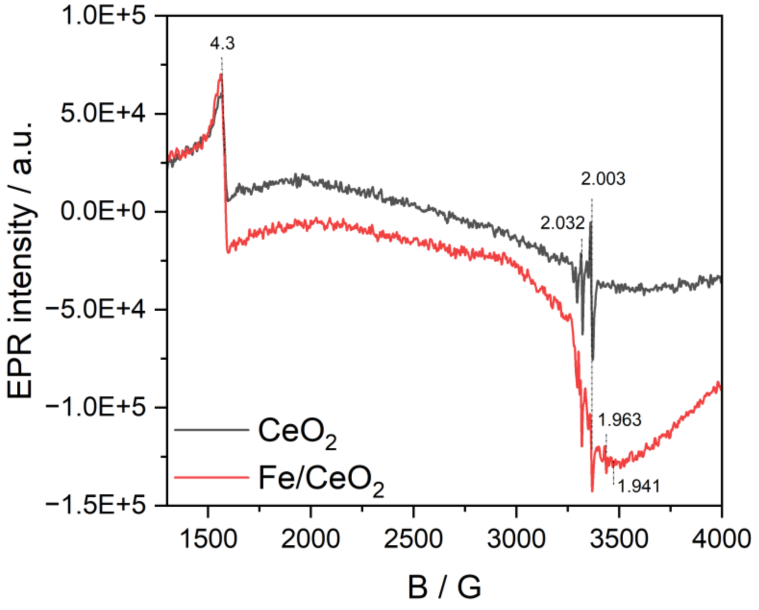

Figure S15. Ex-situ EPR spectra recorded at 25 °C for the ambient air-exposed fresh CeO_2 support and the Fe/CeO_2 catalyst.

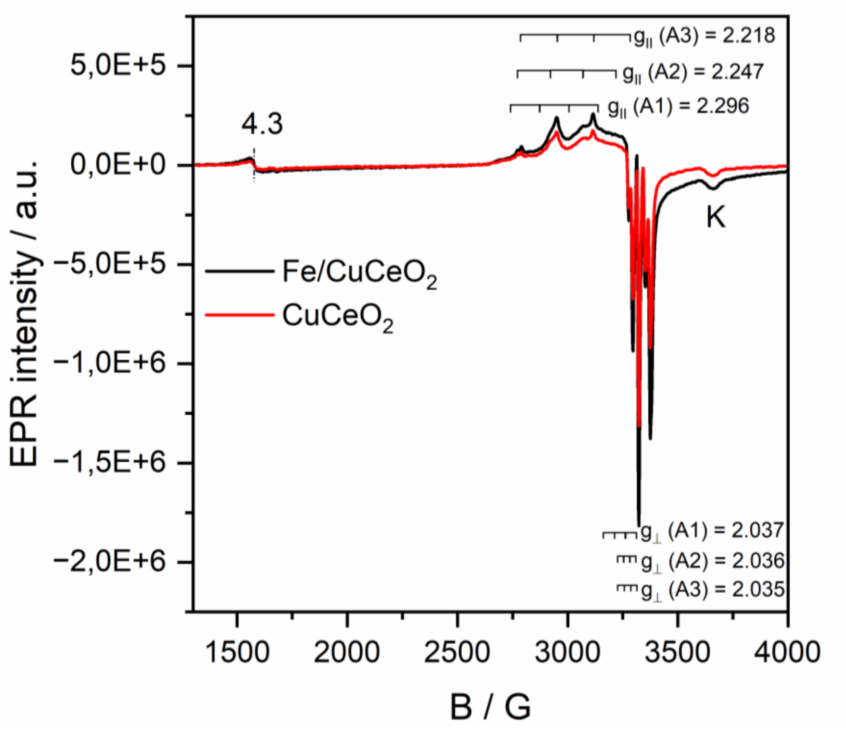

Figure S16. Ex-situ EPR spectra of the fresh $CuCeO_2$ and $Fe/CuCeO_2$ catalysts recorded at 25 °C.

The EPR spectrum of the fresh bare CeO_2 shows some weak signals (**Figure S15**): (1) a signal at g = 2.003, attributed to oxygen vacancies; (2) a set of four narrow lines ($g_{\perp}$ = 2.032), assignable to a tiny Cu^{2+} impurity in the CeO_2 support; (3) a signal at g = 4.3 originating from Fe^{3+} [1] ions present as impurities in the quartz used as diluted material. The Fe/CeO_2 sample (**Figure S15**) exhibits similar signals of oxygen vacancies (g = 2.003) and the set of four narrow lines from Cu impurity in the CeO_2 support. Additionally, there is a signal at g = 4.3 of Fe^{3+} ions, but with higher intensity than that of the support. Furthermore, a superimposed broad signal at g = 2 arises from magnetically interacting Fe species.[1] Moreover, this sample exhibits additional axial signals at $g_{\perp}$ = 1.963 and $g_{||}$ = 1.941. These signals have been assigned to Ce^{3+} ions associated with a defect center (Ce^{3+}-□).[2] The EPR spectra depicted in **Figure S16**, for both the freshly calcined $CuCeO_2$ and Fe/$CuCeO_2$ catalysts reveal the existence of three distinct Cu^{2+} sites. The parameters of these Cu^{2+} EPR signals, obtained through spectral simulation, are detailed in **Table S3**. The predominant axial signals (type A) exhibit a four-line hyperfine structure in both parallel and perpendicular components. This characteristic arises from a magnetic interaction between the spin of the unpaired electron and the nuclear spin of Cu (I= 3/2). Notably, the variations in g and A values among these sites reflect different coordination environments of Cu^{2+} ions, despite their tetragonally distorted octahedral coordination, as evidenced by $g_{||} > g_{\perp} > g_e$ (the free-electron g value, ~2.0023).[3,4] Compared to signal A1, the shifts of $g_{||}$ to lower values and $A_{||}$ to higher values of signals A2 and A3 indicate a change in the Cu^{2+} coordination of signals A2 and A3 to square pyramidal and square planar coordination, respectively. Site A3 is typically located at ceria subsurface positions.[5] The high contribution of signals A2 and A3 to the EPR spectra of both catalysts indicates that most of the Cu sites are near the surface. In addition, a weak signal, denoted as K at 3600 G, has been assigned to Cu^{2+} ion pairs (Cu^{2+} dimer) resulting from the interaction

between unpaired electrons of two equivalent Cu^{2+} ions according to the literature.[6-8] However, unlike the fine structure of seven narrow lines often exhibited in the EPR spectra of Cu^{2+} dimers,[9] this feature in our case is unresolved, and only the perpendicular component can be observed, indicating its location at or near the surface of the catalyst. [6] These Cu^{2+} dimmer species could be responsible for the weak CO adsorption band at 2048 cm^{-1} in the in-situ DRIFT co adsorption spectrum over both $CuCeO_2$ and Fe/ $CuCeO_2$ catalysts. The EPR signal intensity of isolated Cu^{2+} in $CuCeO_2$ is lower than that in Fe/$CuCeO_2$ (**Figure S16**), indicating a higher amount of isolated Cu^{2+} in the latter. Considering that $CuCeO_2$ in both catalysts is from the same batch, this suggests that the addition of Fe stabilizes the single sites of Cu by forming strongly bound singly dispersed Cu-Fe bimetal sites. Moreover, the higher intensity of the signal at g = 4.3 arises from Fe^{3+} single sites, and the presence of a superimposed broad signal around g = 2 in Fe/$CuCeO_2$ indicates the formation of small Fe_2O_3 clusters in this catalyst.

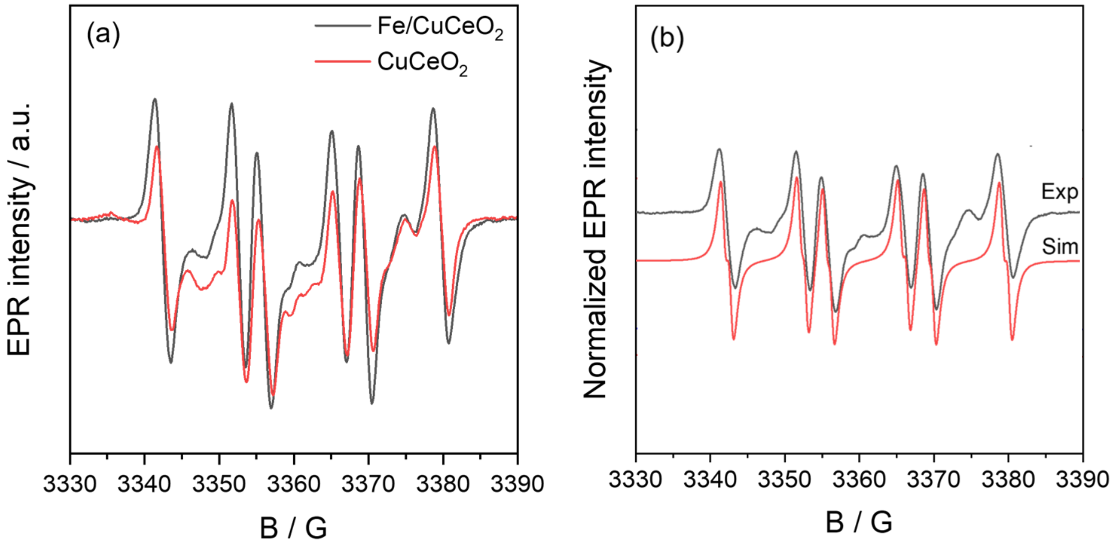

Figure S17. (a) EPR spectra show the formation of DMPO-$O_2^{\bullet-}$ spin adducts after adding a mixture of DMPO/cyclohexane to ambient air-exposed fresh calcined $CuCeO_2$ and Fe/$CuCeO_2$ catalysts. (b) Normalized EPR spectra, together with the corresponding simulated spectra (red lines).

To confirm the role of Fe in the increase in the formation of O-vacancy defects, we performed a surface titration experiment on two fresh catalysts using the DMPO spin trap. The EPR spectra of the two air-exposed fresh catalysts, after adding a mixture of DMPO/cyclohexane at room temperature (**Figure S17**), display the characteristic peaks of the DMPO/$O_2^{\bullet-}$ adducts with the parameters g = 2.0068, A_N = 13.59 G, $A_H{}^{\beta}$ = 10.12 G, $A_H{}^{\gamma}$ = 0.89 G. [8, 10] The superoxide radical $O_2^{\bullet-}$ is formed through the activation of pre-adsorbed gaseous O_2 from ambient air by oxygen vacancies. Therefore, the higher intensity of the DMPO/$O_2^{\bullet-}$ signal in the Fe/CuCeO_2 sample indicates a higher amount of O-vacancy in this sample.

Figure S17a displays the EPR spectra obtained after adding a mixture of DMPO/cyclohexane at room temperature to the two ambient air-exposed fresh calcined catalysts. It reveals the characteristic signals of the DMPO/$O_2^{\bullet-}$ adducts. According to the literature,[8, 10] the simulated signal in **Figure S17b**, with the following parameters: g = 2.0068, A_N = 13.59 G, $A_H{}^{\beta}$ = 10.12 G, $A_H{}^{\gamma}$ = 0.89 G, can be assigned to DMPO-OOH resulting from trapping superoxide with DMPO. The higher intensity of this signal in the Fe/CuCeO_2 catalyst indicates a greater amount of $O_2^{\bullet-}$ radicals formed via the activation of adsorbed gaseous O_2 by oxygen vacancies on its surface.

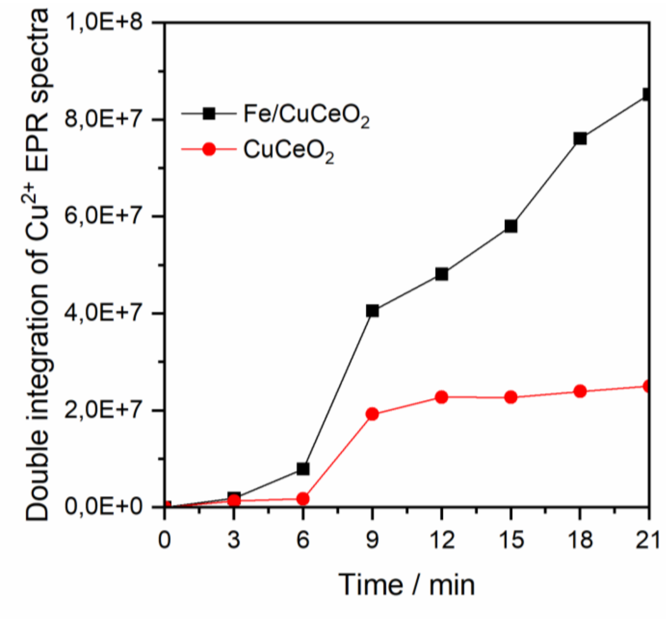

Figure S18. Double integral area of the EPR signals of Cu^{2+} forming under a CO_2 flow at 300 °C over $CuCeO_2$ and $Fe/CuCeO_2$.

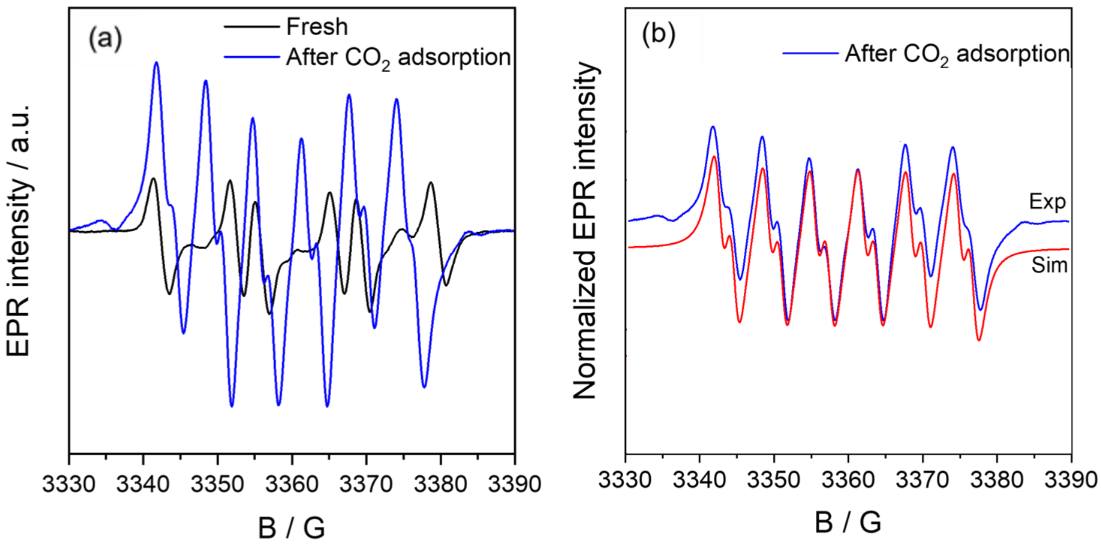

Figure S19. (a) EPR spectra of DMPO spin trap adducts recorded at room temperature after the addition of a DMPO/cyclohexane mixture on CO_2-exposed $Fe/CuCeO_2$ after evacuation and reduction at 300 °C in 100% H_2 (b) Normalized EPR spectrum of (a) after CO_2 adsorption along with the corresponding simulated spectrum (red line). EPR spectrum of DMPO spin trap adducts,

recorded at room temperature after the addition of a DMPO/cyclohexane mixture on an ambient air-exposed fresh Fe/CuCeO_2 catalyst (black spectrum), is also included in (a) to differentiate between DMPO/$O_2^{\bullet-}$ and DMPO-$CO_3^{\bullet-}$ adducts.

The EPR spectrum (blue line) presented in **Figure S19a** was recorded following a series of treatments. Initially, the catalyst underwent evacuation and subsequent reduction at 400 °C in 100% H_2. Subsequently, it was exposed to 100% CO_2, followed by cooling to room temperature, before adding a mixture of DMPO/cyclohexane. In **Figure S19b** (blue line), a new EPR signal is observed, characterized by simulated parameters g = 2.0069, A_N = 12.90 G, $A_H{}^{\beta}$ = 6.48 G, $A_H{}^{\gamma}$ = 1.88 G. These parameters closely resemble those of DMPO-OCO_2 or DMPO-$CO_3^{\bullet-}$ adducts, known to be generated from UV photolysis of H_2O_2 and DMPO in the presence of Na_2CO_3 (or $NaHCO_3$), or DMPO-OR (alkoxy radical adduct of DMPO) formed during in situ heating of solutions of t-butyl hydroperoxide in base oil containing DMPO at 90 °C.[11] Previous theoretical and experimental studies have reported the formation of various modes of $CO_2^{\bullet-}$ addition to DMPO. Therefore, the interaction of CO_2 with the surface of reduced Fe/CuCeO2 results in the creation of $CO_2^{\bullet-}$/$CO_3^{\bullet-}$ radicals.

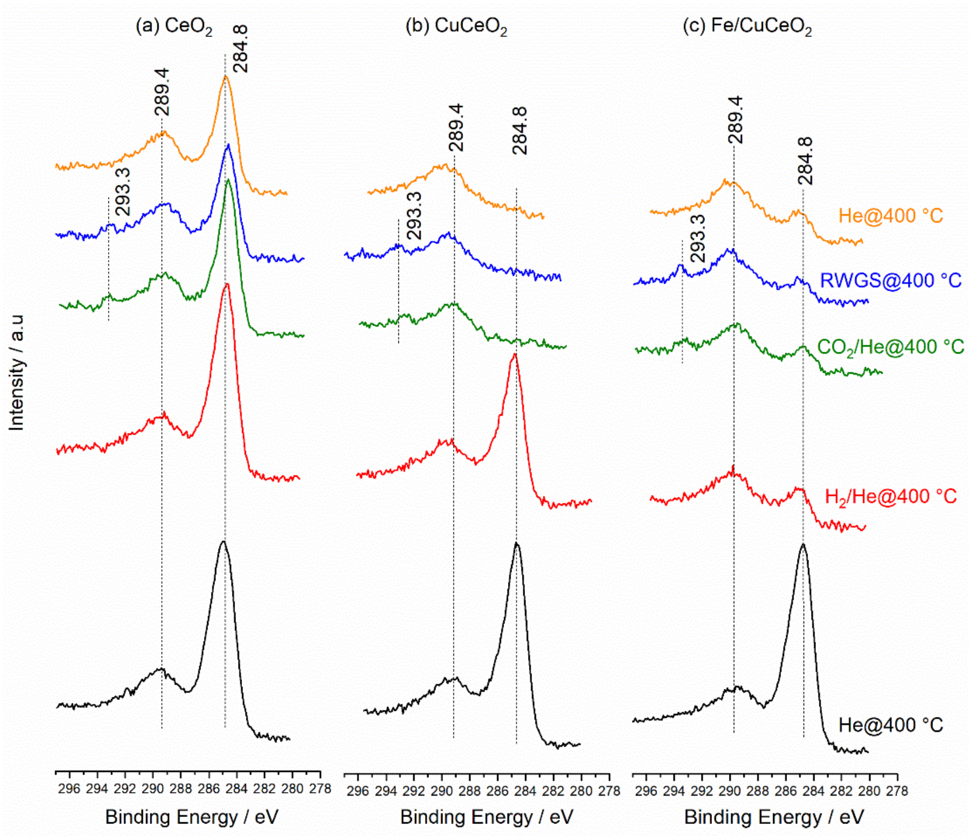

Figure S20. High-resolution XPS spectra of C 1s region recorded during NAP-XPS experiments at the indicated conditions for (a) CeO_2 (b) $CuCeO_2$ and (c) $Fe/CuCeO_2$ catalysts.

Table S1. Structural Properties of the as-synthesized catalysts.

Catalysts	Metal content [a] / wt.%		S_{BET}[b] /$m^2.g^{-1}$	V_p[b]/$cm^3.g^{-1}$	D_p[b]/ nm
	Fe (0.3)	Cu (0.5)			
CeO_2	---	---	98	0.172	5.3
Fe/CeO_2	0.29	---	92	0.166	5.3
$CuCeO_2$	---	0.47	100	0.154	4.7
$Fe/CuCeO_2$	0.31	0.42	91	0.148	5.2

[a] Determined via ICP-OES analysis. Values in parentheses represent the nominal loadings of Fe and Cu metals.

[b] Determined via N_2 adsorption-desorption analysis

Table S2. Structural Properties of the as-synthesized catalysts.

Catalysts	H_2 consumption [a] / μmoles.g_{cat}^{-1}	
	Low-temperature region (150-570 °C) [b]	High-temperature region (> 570 °C)
CeO_2	330	900
Fe/CeO_2	419 (81)	947
$CuCeO_2$	399 (74)	730
$Fe/CuCeO_2$	481 (66^{Cu} + 79^{Fe})	860

[a] The amounts of hydrogen consumption determined via TPR recorded as a function of time (**Figure S4**) were quantified on the basis of the TPR results obtained using known amounts of CuO standard.

[b] Values in Parentheses represent the reduction of Cu and Fe content (based on ICP results) assuming that all the Cu and Fe participate to the reduction changing oxidation state from +2 and +3 to zero.

Table S3. EPR parameters of different Cu related signals from the $CuCeO_2$ and $Fe/CuCeO_2$ catalysts obtained by simulation.

Signal	$g_{\parallel}$	$g_{\perp}$	$A_{\parallel}$ (MHz)	$A_{\perp}$ (MHz)
A1	2.296	2.037	425	77
A2	2.247	2.036	480	49
A3	2.218	2.035	510	57
K		3600G		

References

(1) Vélez, R. P.; Ellmers, I.; Huang, H. M.; Bentrup, U.; Schünemann, V.; Grünert, W.; Brückner, A. Identifying active sites for fast NH_3-SCR of NO/NO_2 mixtures over Fe-ZSM-5 by operando EPR and UV-vis spectroscopy. *J. Catal.* **2014**, *316*, 103-111. DOI: 10.1016/j.jcat.2014.05.001.

(2) Abi-Aad, E.; Bennani, A.; Bonnelle, J.-P.; Aboukaïs, A. Transition-metal ion dimers formed in CeO_2: an EPR study. *J. Chem. Soc., Faraday Trans.* **1995**, *91* (1), 99-104. DOI: 10.1039/ft9959100099.

(3) Rabeah, J.; Stosser, R.; Jiao, H.; Troyanov, S. I.; Radnik, J.; Dwelk, H.; Meisel, M.; Bruckner, A. Tuning the electronic and spin complexity in organic-inorganic molecular hybrid compounds. *Chem. Eur. J.* 20**12**, *18* (21), 6433-6436. DOI: 10.1002/chem.201200931.

(4) Hathaway, B. J.; Billing, D. E. The electronic properties and stereochemistry of mono-nuclear complexes of the copper(II) ion. *Coord. Chem. Rev.* **1970**, *5* (2), 143-207. DOI: 10.1016/S0010-8545(00)80135-6.

(5) A. Mart´ınez-Arias, M. F. a.-G. ı., J. Soria, and J. C. Conesa. Spectroscopic Study of a Cu/CeO_2 Catalyst Subjected to Redox Treatments in Carbon Monoxide and Oxygen. *J. Catal.* **1999**, *182*, 367–377. DOI:10.1006/jcat.1998.2361

(6) Martinez-Arias, A.; Fernandez-Garcia, M.; Soria, J.; Conesa, J. C. Spectroscopic Study of a Cu/CeO_2 Catalyst Subjected to Redox Treatments in Carbon Monoxide and Oxygen. *J. Catal.* **1999**, *182* (2), 367-377. DOI 10.1006/jcat.1998.2361.

(7) Kais, A. A.; Bennani, A.; Aissi, C. F.; Wrobel, G.; Guelton, M. Reduction Effect on Cerium Oxide Catalysts Doped with Copper(II) Ions - an Electron-Paramagnetic Resonance Study. *J. Chem. Soc. Faraday Trans.* **1992**, *88* (9), 1321-1325. DOI:10.1039/ft9928801321.

(8) Mosrati, J.; Abdel-Mageed, A. M.; Vuong, T. H.; Grauke, R.; Bartling, S.; Rockstroh, N.; Atia, H.; Armbruster, U.; Wohlrab, S.; Rabeah, J.; Brückner, A. Tiny Species with Big Impact: High Activity of Cu Single Atoms on CeO_2-TiO_2 Deciphered by Spectroscopy. *ACS Catal.* **2021**, *11* (17), 10933-10949. DOI: 10.1021/acscatal.1c02349.

(9) Wang, F.; Büchel, R.; Savitsky, A.; Zalibera, M.; Widmann, D.; Pratsinis, S. E.; Lubitz, W.; Schüth, F. In Situ EPR Study of the Redox Properties of CuO–CeO_2 Catalysts for Preferential CO Oxidation (PROX). *ACS. Catal.* **2016**, *6* (6), 3520-3530. DOI: 10.1021/acscatal.6b00589.

(10) Clément, J. L.; Ferré, N.; Siri, D.; Karoui, H.; Rockenbauer, A.; Tordo, P. Assignment of the EPR spectrum of 5,5-dimethyl-1-pyrroline -oxide (DMPO) superoxide spin adduct. *J. Org. Chem.* **2005**, *70* (4), 1198-1203. DOI: 10.1021/jo048518z.

(11) Babic, N.; Pondaven, S.; Vezin, H. EPR Spin-Trapping Study of Free Radical Intermediates in Polyalphaolefin Base Oil Autoxidation. *Polym. Degrad. Stabil.* **2021**, *192*. DOI: ARTN 109687

10.1016/j.polymdegradstab.2021.109687.
